# Supplementary material for: Influence of pre-existing multimorbidity on receiving a hip arthroplasty: cohort study of 28 025 elderly subjects from UK primary care
Source: BMJ Open. 2021 Sep 23;11(9):e046713. doi: 10.1136/bmjopen-2020-046713 (PMC8461704; doi:10.1136/bmjopen-2020-046713)
Supplement: Supplementary data [file bmjopen-2020-046713supp001.pdf]

Appendix 1: CPRD GOLD Coverage

The CPRD was established in 1987 and is a not-for-profit research service funded by the NHS National Institute for Health Research (NIHR) and the Medicines and Healthcare products Regulatory Agency (MHRA). It is now owned by the UK Department of Health. CPRD collects data from GP practices using Vision systems, with approximately 718 in total. The nomenclature of the CPRD has evolved over time: originally termed the General Practice Research Database (GPRD), until it was launched as the CPRD in 2012. It is now termed CPRD GOLD, to differentiate it from a recently launched 'sister' database CPRD Aurum that collects data from GP practices using EMIS Web software. Henceforth, use of the term CPRD refers to CPRD GOLD. CPRD contains the records of 17 million patients, of which 4.4 million are active, covering approximately 7% of the UK population (1). Importantly, CPRD patients are broadly representative of the UK population in terms of age, sex, ethnicity and body mass index distribution (2).

1. Clinical Practice Research Datalink. https://www.cprd.com/intro.asp.

2. Herrett E, Gallagher AM, Bhaskaran K, et al. Data Resource Profile: Clinical Practice Research Datalink (CPRD). *Int J Epidemiol*. 2015;44(3):827-836. doi:10.1093/ije/dyv098

Appendix 2: Read codes for hip OA

1. N053512 Hip OA NOS
2. N05z511 Hip OA NOS
3. N05zJ00 OA NOS, of hip
4. Nyu2100 [X]Other primary coxarthrosis
5. N051900 Primary coxarthrosis, bilateral

Appendix 3: Disorders covered by the Quality and Outcomes Framework (QOF).

1. asthma
2. atrial fibrillation
3. hypertension
4. cancer
5. chronic kidney disease
6. COPD
7. Dementia
8. Depression
9. diabetes,
10. epilepsy
11. heart failure
12. learning disability
13. mental health disorder
14. osteoporosis
15. peripheral arterial disease
16. rheumatoid arthritis
17. stroke and TIA
