# Supplementary figures and images for: Influence of pre-existing multimorbidity on receiving a hip arthroplasty: cohort study of 28 025 elderly subjects from UK primary care
Source: BMJ Open. 2021 Sep 23;11(9):e046713. doi: 10.1136/bmjopen-2020-046713 (PMC8461704; doi:10.1136/bmjopen-2020-046713)

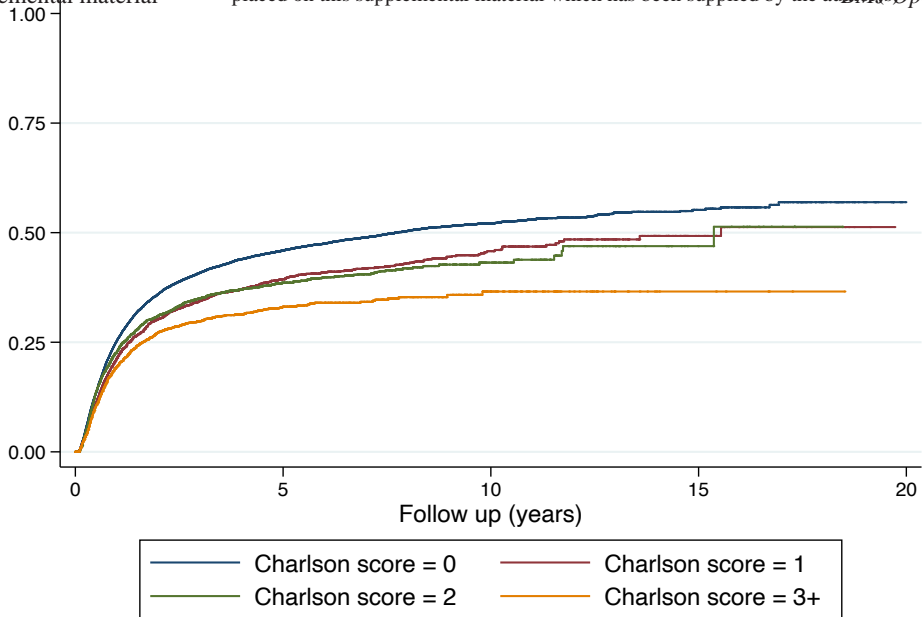

Supplement: Supplementary data [file bmjopen-2020-046713supp002.pdf]

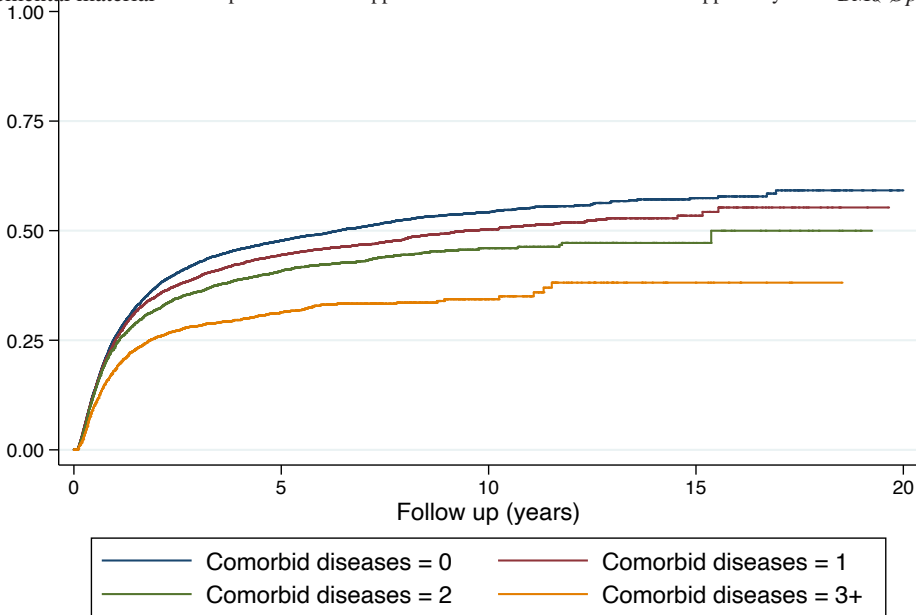

Supplement: Supplementary data [file bmjopen-2020-046713supp003.pdf]

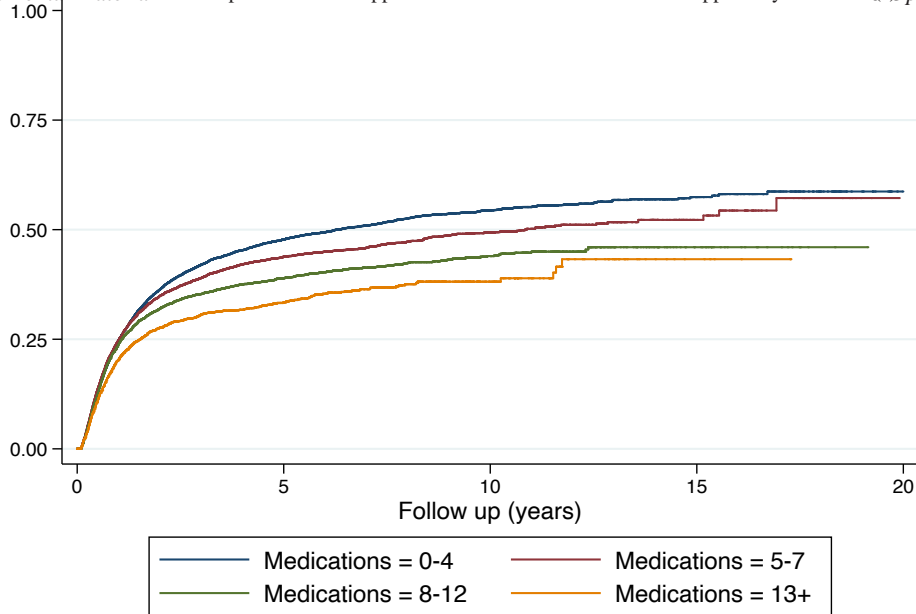

Supplement: Supplementary data [file bmjopen-2020-046713supp004.pdf]

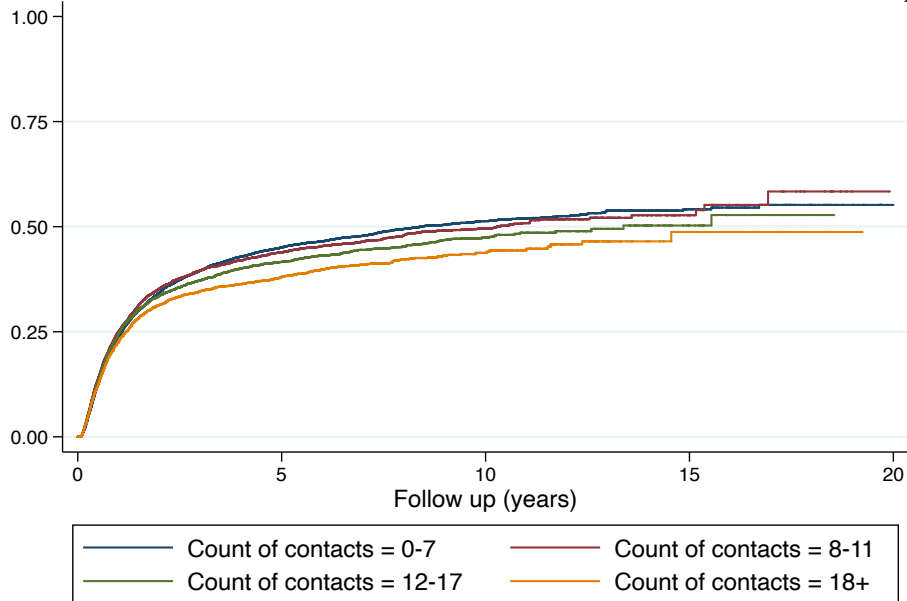

Supplement: Supplementary data [file bmjopen-2020-046713supp005.pdf]

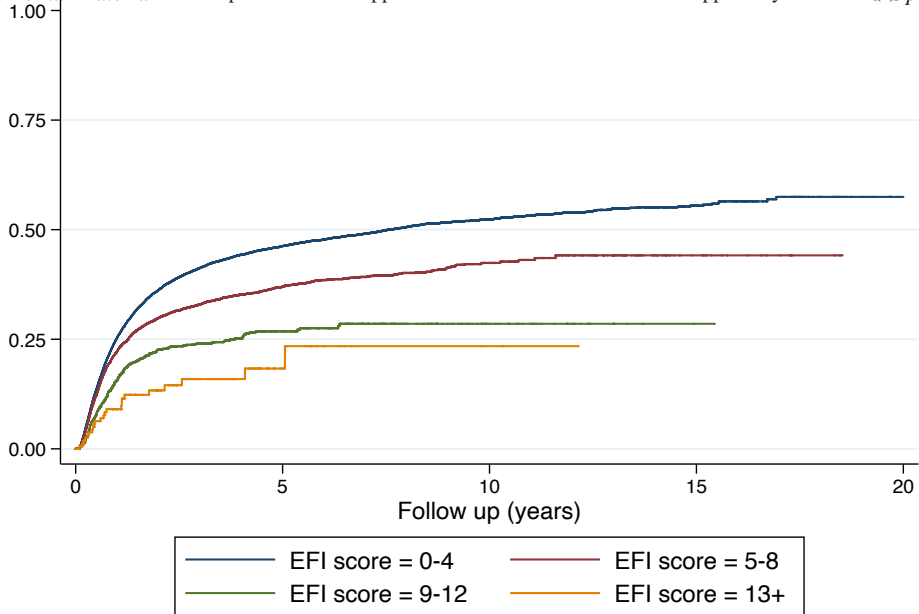

Supplement: Supplementary data [file bmjopen-2020-046713supp006.pdf]
