# Supplementary material for: Influence of pre-existing multimorbidity on receiving a hip arthroplasty: cohort study of 28 025 elderly subjects from UK primary care
Source: BMJ Open. 2021 Sep 23;11(9):e046713. doi: 10.1136/bmjopen-2020-046713 (PMC8461704; doi:10.1136/bmjopen-2020-046713)
Supplement: Supplementary data [file bmjopen-2020-046713supp007.pdf]

Supplementary Table 1: Mean time to surgery by morbidity category

	**Category**	**Mean time to surgery (years)**
Charlson Comorbidity Index	0	1.46
	1	1.52
	2	1.19
	3+	1.10
Count of chronic diseases	0	1.57
	1	1.40
	2	1.28
	3+	1.10
Count of medications prescribed	0-4	1.60
	5-7	1.36
	8-12	1.21
	13+	1.17
Count of contacts with primary care	0-7	1.52
	8-11	1.40
	12-17	1.31
	18+	1.25
Electronic Frailty Index	0-4	1.49
	5-8	1.18
	9-12	0.99
	13+	1.12

Supplementary Table 2: THA surgery rates by Morbidity Status based on HES recorded surgery

**Morbidity scale**	**Category**	Total at risk	Competing events	THR events	Unadjusted HR (95% CI)	Adjusted HR* (95% CI)
Charlson Comorbidity Index	0	10,980	1,992	4,552	Ref	Ref
	1	2,037	584	703	0.79 (0.73-0.86)	0.85 (0.78-0.92)
	2	2,034	466	707	0.86 (0.79-0.93)	0.85 (0.78-0.92)
	3+	1,732	495	525	0.72 (0.65-0.78)	0.68 (0.62-0.75)
Count of chronic diseases	0	5,559	1,059	2,412	Ref	Ref
	1	5,280	1,069	2,150	0.97 (0.91-1.03)	0.93 (0.88-0.98)
	2	3,306	724	1,182	0.86 (0.80-0.92)	0.81 (0.76-0.87)
	3+	2,638	685	743	0.68 (0.63-0.74)	0.63 (0.58-0.69)
Count of medications	0-4	6,192	1,138	2,695	Ref	Ref
	5-7	4,445	941	1,759	0.94 (0.89-1.00)	0.94 (0.89-1.00)
	8-12	4,188	943	1,474	0.85 (0.80-0.90)	0.83 (0.78-0.89)
	13+	1,958	515	559	0.70 (0.64-0.76)	0.66 (0.60-0.73)
Count of contacts with primary care	0-7	7,004	1,389	2,896	Ref	Ref
	8-11	3,817	734	1,473	0.96 (0.90-1.02)	0.95 (0.89-1.01)
	12-17	3,194	684	1,186	0.93 (0.87-0.99)	0.92 (0.85-0.98)
	18+	2,968	730	932	0.84 (0.78-0.90)	0.83 (0.77-0.89)
Electronic Frailty Index	0-4	11,318	2,239	4,793	Ref	Ref
	5-8	4,426	986	1,468	0.82 (0.77-0.87)	0.78 (0.73-0.83)
	9-12	920	266	209	0.55 (0.48-0.64)	0.54 (0.47-0.63)
	13+	119	46	17	0.35 (0.22-0.57)	0.34 (0.21-0.55)
**Total**		16,783	3,537	6,487		
