# Supplementary material for: Influence of pre-existing multimorbidity on receiving a hip arthroplasty: cohort study of 28 025 elderly subjects from UK primary care
Source: BMJ Open. 2021 Sep 23;11(9):e046713. doi: 10.1136/bmjopen-2020-046713 (PMC8461704; doi:10.1136/bmjopen-2020-046713)
Supplement: Author's manuscript [file bmjopen-2020-046713.draft_revisions.pdf]

BMJ Open is committed to open peer review. As part of this commitment we make the peer review history of every article we publish publicly available.

When an article is published we post the peer reviewers' comments and the authors' responses online. We also post the versions of the paper that were used during peer review. These are the versions that the peer review comments apply to.

The versions of the paper that follow are the versions that were submitted during the peer review process. They are not the versions of record or the final published versions. They should not be cited or distributed as the published version of this manuscript.

BMJ Open is an open access journal and the full, final, typeset and author-corrected version of record of the manuscript is available on our site with no access controls, subscription charges or pay-per-view fees (http://bmjopen.bmj.com).

If you have any questions on BMJ Open's open peer review process please email info.bmjopen@bmj.com

BMJ Open

Influence of pre-existing multimorbidity on receiving a hip arthroplasty: Cohort study of 28,026 elderly subjects from UK primary care

Journal:	*BMJ Open*
Manuscript ID	bmjopen-2020-046713
Article Type:	Original research
Date Submitted by the Author:	11-Nov-2020

Complete List of Authors:	Ferguson, Rory; University of Oxford, NDORMS<br>Prieto-Alhambra, Daniel; University of Oxford, Centre for Statistics in Medicine, NDORMS<br>Peat, George; Keele University, Research Institute for Primary Care & Health Sciences<br>Delmestri, Antonella; University of Oxford, Nuffield Department of Orthopaedics, Rheumatology, and Musculoskeletal Sciences<br>Jordan, Kelvin; Keele University, School of Primary, Community and Social Care<br>Strauss, Vicky; University of Oxford, Centre for Statistics in Medicine, Ndorms<br>Valderas, Jose; University of Exeter Medical School,<br>Walker, Christine; Keele University, Research User Group, Arthritis Research UK Primary Care Centre, Research Institute of Primary Care and Health Sciences<br>Yu, Dahai; Keele University, Research Institute for Primary Care & Health Sciences<br>Glyn-Jones, Sion; University of Oxford<br>Silman, Alan; University of Oxford Nuffield Department of Orthopaedics Rheumatology and Musculoskeletal Sciences, Botnar Institute

Keywords:	Hip < ORTHOPAEDIC & TRAUMA SURGERY, Health policy < HEALTH SERVICES ADMINISTRATION & MANAGEMENT, SURGERY, GERIATRIC MEDICINE

SCHOLARONE™
Manuscripts

BMJ

I, the Submitting Author has the right to grant and does grant on behalf of all authors of the Work (as defined in the below author licence), an exclusive licence and/or a non-exclusive licence for contributions from authors who are: i) UK Crown employees; ii) where BMJ has agreed a CC-BY licence shall apply, and/or iii) in accordance with the terms applicable for US Federal Government officers or employees acting as part of their official duties; on a worldwide, perpetual, irrevocable, royalty-free basis to BMJ Publishing Group Ltd ("BMJ") its licensees and where the relevant Journal is co-owned by BMJ to the co-owners of the Journal, to publish the Work in this journal and any other BMJ products and to exploit all rights, as set out in our licence.

The Submitting Author accepts and understands that any supply made under these terms is made by BMJ to the Submitting Author unless you are acting as an employee on behalf of your employer or a postgraduate student of an affiliated institution which is paying any applicable article publishing charge ("APC") for Open Access articles. Where the Submitting Author wishes to make the Work available on an Open Access basis (and intends to pay the relevant APC), the terms of reuse of such Open Access shall be governed by a Creative Commons licence – details of these licences and which Creative Commons licence will apply to this Work are set out in our licence referred to above.

Other than as permitted in any relevant BMJ Author's Self Archiving Policies, I confirm this Work has not been accepted for publication elsewhere, is not being considered for publication elsewhere and does not duplicate material already published. I confirm all authors consent to publication of this Work and authorise the granting of this licence.

For peer review only

Influence of pre-existing multimorbidity on receiving a hip arthroplasty: Cohort study of 28,026 elderly subjects from UK primary care

RJ Ferguson [1]
D Prieto-Alhambra [1]
G Peat [2]
A Delmestri [1]
KP Jordan [2]
V Strauss [1]
JM Valderas [3]
C Walker [2]
D Yu[2]
S Glyn-Jones [1]
AJ Silman[1]

[1]Nuffield Department of Orthopaedics, Rheumatology and Musculoskeletal Sciences, University of Oxford, Oxford, UK.
[2]Primary Care and Health Sciences Department, Keele University, Keele, UK.
[3]University of Exeter Medical School, Exeter, UK.
[4]Oxford University Hospital NHS Foundation Trust, Oxford, UK.

Correspondence to Professor Silman
Nuffield Department of Orthopaedics, Rheumatology and Musculoskeletal Sciences, University of Oxford
Botnar Research Centre, Windmill Road
Headington, Oxford, OX3 7LD.
alan.silman@ndorms.ox.ac.uk

Word count: abstract 271, main paper 2642

Key words: Hip osteoarthritis, arthroplasty, multimorbidity, health care, health service

Acknowledgements

We acknowledge Professor Andy Judge for advice on statistical analysis at the earliest stages of this project . We are also grateful to Dr Andrew Clegg, and Stephen Pye for guidance in the use of the electronic frailty index. Dr John Griffiths and Professor Nigel Arden, provided very helpful advice at the design stage on the challenges of anaesthetic assessment and on studying osteoarthritis from an epidemiological perspective..

For peer review only

Abstract

Objective

To evaluate how pre-existing levels of comorbidity influence the likelihood of receiving a total hip arthroplasty (THA).

Design

Longitudinal record linkage study

Setting

UK national sample from primary care linked to their secondary care records

Participants

All 28,025 patients, newly recorded as having hip osteoarthritis identified from the national primary care register CPRD which allowed extraction of their background health and morbidity status using 5 different constructs: Charlson Comorbidity Index (CCI), Electronic Frailty Index and counts of chronic disorders (from list of 17), prescribed medications and number of primary care visits prior to the recording of osteoarthritis.

Outcome measures

Any mention of having received a THA was extracted from the primary care record and the linked secondary care database: HES (Hospital Episode Statistics)

Results

In all, 40% had THA over a follow up to 17 years. All approaches to assessing comorbidity were associated with a reducing likelihood of THA with increasing level of poor pre-operative health. This reduction was observed, even at low levels of comorbidity not associated with important increased peri-operative risk. For examples the adjusted hazard ratio for THA in the only mildly frail group was 0.77 (0.74-0.81) and in those with a CCI score of just 1 was 0.84 (0.79-0.89) compared to those in the healthiest categories

Conclusion

Markers of pre-existing poor health, not unexpectedly, will influence decision for THA in an elderly person with end stage hip osteoarthritis. However, evidence of this selection is present even in people with moderate decrements in their health, despite our having shown from the same national data sets that the balance of benefits to risk in these individuals was positive

(271 words)

For peer review only

Strengths and Limitations of this study

- National large sample of older patients recorded in primary care with newly reported osteoarthritis of the hip
- Multi-dimensional approach to assessing their concurrent morbidity and health status
- Linkage to how the above influences their subsequent likelihood of having a hip replacement
- Such study using routine national datasets have advantage of size, access to health record and linkage
- Inevitable but challenge to quantify completeness and accuracy of diagnostic and treatment records

For peer review only

Introduction

Total hip arthroplasty (THA) is a highly cost-effective procedure for hip osteoarthritis (OA)(1). The mean age at surgery in many OECD countries is currently around 70 (2), and consequently many patients being considered for THA will have multiple comorbidities (3). The latter raises concerns about operative risk and hence deter surgery thereby limiting access to those who may otherwise benefit. Balancing risks and benefits of surgical procedures, for examples invasive cardiac procedures, has been well discussed for such potentially life threatening disorders (4). There is however very little evidence to guide health care professionals on the risks and benefits of THA in elderly patients with multimorbidity but who have severe limitation of quality of life (5-6). In one report recently published from a tertiary care centre from the USA, background comorbidity was linked to both an increased risk of complications and a poorer outcome (7). The concern, though, is that those with relatively modest background levels of multimorbidity may be excluded from surgery. Our objective was to determine, using a national primary care database, how levels of pre-existing multimorbidity influence the likelihood of receiving THA.

Methods

Design

In brief we undertook a cohort study analysing routinely collected data gathered from the UK's Clinical Practice Research Datalinks (CPRD) (8). This longitudinal primary care record of elderly (patients aged 65 and over) was interrogated to identify patients with a first recorded diagnosis of hip OA. From these individuals, data from their CPRD record was used to extract information on their general health and disease status from which 5 approaches to scoring their comorbidity were derived. Patients were then followed up to identify those recorded as receiving a THA.

Subjects

These were 28,025 patients who had been diagnosed with hip OA in CPRD GOLD records between January 1st 1995 and April 30th 2014. The diagnosis of hip OA was based on being recorded with at least one from a list of relevant READ codes (Supplementary File 1) (9). A validation exercise, obtaining clinical and radiographic data on a small subsample of these patients showed a sufficiently high level of diagnostic accuracy against accepted clinical criteria (10).

Multimorbidity markers

Information extracted from the longitudinal primary care records was used to score patients into different categories of 5 separate approaches to assessing their background morbidity and health at the time of recording of hip OA. The spectrum of measures aimed to cover (i) specific risk scores accepted as predictive for post-surgical morbidity, (ii) more generalised

For peer review only

scores of health and function and (iii) scores of health care utilisation. The specific scores used were:

1) Simple count of ever mention of the 17 chronic disorders included in the Quality and Outcomes Framework (QOF) (11) (Supplementary File 2)– a scheme to ensure maximal primary care compliance with recording chronic disorders
2) Charlson Comorbidity Index (CCI) based on the cumulative burden of its components over the previous 5 years. The CCI is a well-accepted measure of peri-and post-operative morbidity (12)
3) Electronic Frailty Index (eFI), a validated tool that aims to score frailty based on the number of different 'deficits' mentioned in the primary care record across a large number of health domains (13).
4) Count of the number of different individual medications in the previous 12 months via CPRD GOLD prescription data
5) Count of consultation events with primary care in the previous 12 months as a measure of health services burden

Follow up

Patients were then followed up using their CPRD record to identify those where there was a record of receiving THA based on relevant codes (14) by January 2015. As recording of THA may not be complete in CPRD, we also considered the alternative source ascertainment of THA based on linkage of CPRD records to the national Hospital Episode Statistic (HES) dataset. HES linkage is only available for England not all general practices in CPRD permitted linkage. We therefore conducted a sensitivity analysis (see below) using the HES data.

Analysis

The scores for each of the five measures of multimorbidity were split into 4 categories. The eFI has 4 categories defined by the developers: fit (0-4), mild frailty (5-8), moderate frailty (9-12), and severe frailty (13+). The actual CCI scores were used to split into categories of score of 0, 1, 2, and 3+. Based on the distribution of the data the counts of number of chronic diseases were also split in categories of score of 0, 1, 2, and 3+. The categories for the number of medications prescribed and of primary care visits were derived from the observed distributions of the actual data, aiming for equal sized quarters: with medication counts divided into scores of 0-4, 5-7, 8-12, and 13+ and number of primary care visits into 0-7, 8-11, 12-17 and 18+. The lowest category for each score was used as the referent category in the analysis.

The analysis was directed towards assessing the relationship between the different categories of each of the multimorbidity scoring systems. Thus, we assessed both the cumulative incidence and the time to event of receiving a THA following the diagnosis of hip OA in the primary care record. There was a concern that the diagnostic label of hip OA may

have been applied once the decision to refer for surgery had been made and an arbitrary decision was thus made to exclude those who had surgery within 30 days from the diagnosis date. For each of the five approaches to scoring morbidity, patients were divided into 4 categories of increasing severity. The overall incidence of THA per 100 patient years was calculated for each category. The association between multimorbidity categories and the likelihood of THA was then investigated using Fine and Gray competing risk analysis models (15) with death being considered a competing event. The models are presented as morbidity-specific hazard ratios (HRs) with 95% confidence intervals (CIs). The lowest multimorbidity category was defined as the referent category. Univariate and then multivariate models- adjusted for age, sex, region of the UK and calendar year of diagnosis, were conducted. We addressed the potential bias of the incompleteness of THA in the CPRD record we undertook a sensitivity analysis in the sub set of practices with HES linkage using that latte as a source of THA

Patient and Public Involvement

This research was funded by the NIHR Research for Patient Benefit scheme which has an absolute requirement that there is patient and public involvement in all relevant stages of the research. The research question had been originally raised in a "Priority Setting Partnership for Priorities for Research in Hip and Knee Arthroplasty" and this was followed by a survey of members of the Keele PPI panel: Research Users Group (RUG). We then tested the suggested questions with the group and received very positive feedback. One of the RUG members (Author CW), herself a patient with direct experience of the target of the research, then became an active member of the research team, participated in all the meetings and advised on the questions that should be asked during the analysis stage. As part of the dissemination phase, Keele PPI group organised a round table event attended by the lead authors where the results were discussed and guidance given on how those members of the public who were present wished to see disseminated

Results

In all 28,025 patients had been diagnosed with hip OA in their CPRD GOLD records between January 1st 1995 and April 30th 2014. Their mean age was 75 and 62% were female. The details of their demographic characteristics are shown in Table 1.

The distributions of morbidity data using the allocation of categories for the patients, at the time of first recording of their hip OA, are shown in Table 2. The CCI scores showed that two thirds had a score of 0, a similar proportion to those who had one or less other chronic disorder. The proportion of subjects in each category of increasing score, showed broadly similar trends for the three 'count' approaches of assessing morbidity. The frailty scores

For peer review only

based on the eFI showed that only around 6 percent could be categorised as being 'moderately' or 'severely' frail.

On follow up 11,413 subjects had a THA at some stage (Figure). As discussed above, we excluded 465 who had surgery within 30 days from the diagnosis date. Of the remainder, 10,948 (39.7%) received a THA during the follow up period with a median interval of just under 10 months, range 1 month to 17 years. The overall incidence of THA during the period of observation was 11.5/100 person years, ie just over 10% per year. The THA rates by morbidity category are shown in Table 3. In this crude analysis, there was very little influence of morbidity on whether or not a patient had surgery during follow up apart from the lower rate in the very small number on the most frail category

Analysis of time to surgery, allowing for deaths during follow up are shown in Table 4, both unadjusted and then adjusted for age, gender, region and year of diagnosis. These results show a marked trend of decreasing likelihood of surgery with increasing levels of morbidity, however defined. Although this was not unexpected, the reduction in surgery rates was observed even in those with the least increase in their morbidity status above 'baseline. Thus, for example, those who were only 'mildly' frail or had a CCI of 1 were 23% and 16% less likely respectively to have surgery. Adjustment for age and gender made little difference to the results suggesting that, it was the morbidity and not age that explains the data

Discussion

This paper describes provides a snapshot of the levels of background morbidity and health utilisation on large national cohort of elderly patients newly presenting to their general practitioner with hip OA. Not surprisingly given concerns about operative risk, the results showed that the greater the pre-existing morbidity the longer the time to surgery. However, this influence on time to surgery is observed even in those with a low surgical risk. In this analysis those who were just mildly frail had almost a quarter lower rate of surgery. Whether comorbidity should influence decision for elective hip surgery has been debated given the relative lack of influence on long term mortality (16). Further, in a companion paper examining the influence of comorbidity on the outcome of THA in the same UK primary care derived population, the benefits of surgery in terms of quality of life These benefits outweighed a modest increased mortality risk were similar independent of baseline morbidity (17 submitted for publication).

Strengths and limitations

The population studied was derived from a national population base, CPRD GOLD, and provided, a representative sample of patients in primary care with OA of the hip (8). A primary care data set is also the only option for providing information on the levels of morbidity at the time of recording the hip diagnosis. These could then be analysed

For peer review only

prospectively to evaluate the relationship between morbidity and the likelihood of surgery. Comorbidity is a concept with no single measure that is perfect (18). We used a broad range of measures, each identifying a different concept of comorbidity that might have influenced surgical decision making.

There are a number of limitations that must be considered before interpreting the results further.

These data are based on the primary care recording of OA of the hip in this electronic dataset. There were likely to be errors both in terms of under-reporting of all cases and diagnostic inaccuracy in those who were included. We (DY, GP and KJ) have previously reported on a retrospective analysis of the presence of codes for hip OA specifically, and OA in general, in patients who had a THA recorded in CPRD (19). In that analysis there was substantial under-reporting of hip OA when relying on a specific code of hip OA, although the under-recording was lower when allowing the primary care to mention OA in any joint. The latter would have been too non-specific for the current analysis. By contrast (as mentioned above) the level of diagnostic inaccuracy in those who were recorded as having hip OA, based on a detailed clinical review of a sample of cases was low (10).

The major aim of the analysis was to study the delay (time to), rather than the fact of, surgery following the recording of the diagnosis of hip OA. Patients attending their primary care physician with hip pain or other relevant symptom may have only been recorded as having OA once a decision to refer for surgery had been made. Indeed, given the potential for under-reporting of OA of the hip, it is perhaps more likely that the OA label may have been made on those with more definite and severe OA.

These points illustrate the challenges in relying on administrative databases from primary care to identify a complete population of cases. Thus, our results on the relationship between comorbidity and THA can only refer to those with a hip OA code.

We are unable to separate from our data set the contribution that baseline comorbidity made between the decision by *primary care to refer for an orthopaedic opinion* from that made in *secondary care as to whether to have surgery*. Although in practice such a separation may be hard to interpret as the referring primary care physician may influence both the patient and the orthopaedic surgeon in their decision.

Any study such as this reflects the decisions made in relation to factors operating in the UK health system at that point in time. Views about the need for surgery and secular changes in resource availability might have influenced our results. We therefore adjusted for year of diagnosis which made no difference. However, it is interesting to see for example how the

For peer review only

recent CoVid-19 epidemic might influence surgical decision making currently. Indeed, the CCI is being modified to collect such information (20).

Comorbidity as assessed by our scores is not static. We examined the influence of the scores at the time of first recording of hip OA. Apart from the health care utilisation type scores (drug counts and primary care visits), the scores were based on accumulation of comorbidities (over 5 years for CCI and ever for eFI and disease count) to the time of diagnosis. Given the median time between primary care recording and surgery was under a year, we suggest that these scores are unlikely to have changed to any important extent

We used CPRD GOLD as our source of data on THA which covers surgery in primary care NHS. In the NHS, data are also available from Hospital Episode Statistics (HES) which can be linked to CPRD. However, HES does not cover Scottish, Northern Ireland or Welsh patients and also linkage to HES was not available for all practices; a link was only possible for just under 17,000 of our cohorts. We did undertake a sensitivity analysis (data not provided) using that subset. The morbidity distributions were similar as were the relationships with the likelihood of surgery

Finally, there are key confounders, which might explain the results. Thus, both BMI and smoking may be key contributors to the decision as to whether a patient with OA has surgery. CPRD GOLD records are incomplete in regard to these variables and there is also concern of over adjustment. BMI might be the major predictor of the likelihood of surgery and should be examined. BMI data were available in 22344 (79.8%) of the cohort but at varying times in relation to the record of OA. There was, however, only a very modest relationship between BMI category and surgery (data not shown) and did not alter the associations with morbidity in that subset.

In conclusion modest background morbidity does influence who has THA in the UK, despite evidence that the harms in such groups outweigh the benefits

For peer review only

Contributorship Statement:
The study was conceived by AJS with SG-J and DP-A and detailed protocol produced by these authors with input from GP, KPJ, JMV and DY. AD was responsible for extracting, preparing and ensuring the quality of the datasets for analysis; analysis was undertaken by RJS supported by VS, DP-A and AJS, CW provided the public and patient input into the design, approach to analysis and interpretation of the data. All authors contributed to the discussion of the results. The manuscript was prepared by AJS and RJF and reviewed by all authors

Competing Interests
None of the authors have any competing interests to report

Funding
This report is independent research funded by the National Institute for Health Research (Research for Patient Benefit programme, PB-PG-0815-20024). The views expressed in this publication are those of the authors and not necessarily those of the NIHR or the Department of Health and Social Care

This study is based in part on data from the Clinical Practice Research Datalink obtained under licence from the UK Medicines and Healthcare products Regulatory Agency. The data is provided by patients and collected by the NHS as part of their care and support. The interpretation and conclusions contained in this study are those of the author/s alone
HES Data/ONS Data shown are Copyright © 2016 are re-used with the permission of The Health & Social Care Information Centre. All rights reserved.
The OPCS Classification of Interventions and Procedures, codes, terms and text is Crown copyright (2016) published by Health and Social Care Information Centre, also known as NHS Digital and licensed under the Open Government Licence available at www.nationalarchives.gov.uk/doc/open-government-licence/open-government-licence.htm.

Data Statement: The data that support the findings of this study are available from CPRD but restrictions apply to the availability of these data, which were used under license for the current study, and so are not publicly available. The datasets extracted from CPRD and used in the analysis during the current study are available from the corresponding author on reasonable request and consistent with the permission received from CPRD

Ethical Approval
No ethical approval was required and anonymised data were provided by as above. No individual patient consent was therefore required

Consent for publication: not applicable

For peer review only

References

1. NHS Digital. Provisional Quarterly Patient Reported Outcome Measures (PROMs) in England. 2017. https://digital.nhs.uk/catalogue/PUB23060.
2. National Joint Registry. National Joint Registry for England, Wales, Northern Ireland and the Isle of Man: 15th Annual Report. 2018.
3. The Academy of Medical Sciences. Multimorbidity: a priority for global health research. 2018 https://acmedsci.ac.uk/file-download/82222577 (accessed April 4, 2019).
4. Orkaby AR, Forman DE. Assessing Risks and Benefits of Invasive Cardiac Procedures in Patients with Advanced Multimorbidity. *Clin Geriatr Med*. 2016;32(2):359-371. doi:10.1016/j.cger.2016.01.004
5. Gordon M, Paulsen A, Overgaard S, Garellick G, Pedersen AB, Rolfson O. Factors influencing health-related quality of life after total hip replacement--a comparison of data from the Swedish and Danish hip arthroplasty registers. *BMC Musculoskelet Disord*. 2013;14:316. Published 2013 Nov 6. doi:10.1186/1471-2474-14-316
6. Greene ME, Rolfson O, Gordon M, Garellick G, Nemes S. Standard Comorbidity Measures Do Not Predict Patient-reported Outcomes 1 Year After Total Hip Arthroplasty. *Clin Orthop Relat Res*. 2015;473(11):3370-3379. doi:10.1007/s11999-015-4195-z
7. Mannion AF, Nauer S, Arsoy D, Impellizzeri FM, Leuning M.The Association Between Comorbidity and the Risks and Early Benefits of Total Hip Arthroplasty for Hip Osteoarthritis J Arthroplasy 2020 (in press) doi.org/10.1016/j.arth.2020.04.090
8. Herrett E, Gallagher AM, Bhaskaran K, *et al.* Data Resource Profile: Clinical Practice Research Datalink (CPRD). *Int J Epidemiol* 2015; **44**: 827–36.
9. https://digital.nhs.uk/services/terminology-and-classifications/read-codes
10. Ferguson RJ, Prieto-Alhambra D, Walker C, et al. Validation of hip osteoarthritis diagnosis recording in the UK Clinical Practice Research Datalink. *Pharmacoepidemiol Drug Saf*. 2019;28(2):187-193. doi:10.1002/pds.4673
11. NHS Primary Care Commissioning *QOF implementation business rules: v11*. http://www.primarycarecontracting.nhs.uk/145.php
12. Charlson ME, Pompei P, Ales KL, MacKenzie CR. A new method of classifying prognostic comorbidity in longitudinal studies: development and validation. *J Chronic Dis* 1987; **40**: 373–83
13. Clegg A, Bates C, Young J, *et al.* Development and validation of an electronic frailty index using routine primary care electronic health record data. *Age Ageing* 2016; **45**: 353–60.
14. Royal College of Surgeons of England, Academy of Medical Royal Colleges. Hospital Episode Statistics and revalidation.2013, RCS England
15. Fine JP, Gray RJ. A proportional hazards model for the subdistribution of a competing risk. J Am Stat Assoc. 1999;94:496-509.
16. Bülow E, Rolfson O, Cnudde P et al. Comorbidity does not predict long-term mortality after total hip arthroplasty. *Acta Orthop*. 2017;88(5):472-477.
17. Fergsuon submitted
18. Mehta HB, Dimou F, Adhikari D, et al. Comparison of Comorbidity Scores in Predicting:10.1097/MLR.0000000000000465

19. Yu D, Jordan KP, Peat G. Under-recording of osteoarthritis in United Kingdom primary care electronic health record data. *Clin Epidemiol*. 2018;10:1195-1201. Published 2018 Sep 12. doi:10.2147/CLEP.S160059
20. https://www.mdcalc.com/charlson-comorbidity-index-cci

For peer review only

Table 1: Demographic Characteristics of Cohort

Variable	Category	N (28025)	%
**Age**	65-69	7200	25.7
	70-74	7229	25.8
	75-79	6421	22.9
	80-84<br>85-89	4233<br>2161	15.1<br>7.7
**Gender**	Male	10,662	38.0
	Female	17,363	62.0
**BMI***	<18.5	257	1.2
	18.5-<25	6,611	29.6
	25-<30	9,569	42.8
	30-<35	4,269	19.1
	35+	1,638	7.3
**IMD quintiles**	Least deprived	4,668	25.9
	2	4,503	25.0
	3	3,981	22.1
	4	3,190	17.7
	Most deprived	1,696	9.4
*no record of BMI in 5681<br>**IMD missing in 9987 as only available on ONS perfect matches and only in England			

For peer review only

Table 2 Background Morbidity Status of Cohort

**Morbidity scale**	**Category**	**N** 28,025	**%**
Charlson Comorbidity Index	0	18,631	66.5
	1	3,245	11.6
	2	3,312	11.8
	3+	2,837	10.1
Count of chronic diseases	0	9,398	33.5
	1	8,957	32.0
	2	5,414	19.3
	3+	4,256	15.2
Count of medications prescribed	0-4	10,392	37.1
	5-7	7,365	26.3
	8-12	6,990	24.9
	13+	3,278	11.7
Count of contacts with primary care	0-7	11,945	42.6
	8-11	6,378	22.8
	12-17	5,212	18.6
	18+	4,490	16.0
Electronic Frailty Index	0-4	19,441	69.4
	5-8	7,005	25.0
	9-12	1,411	5.0
	13+	168	0.6

For peer review only

Table 3. Crude Incidence of Total Hip Arthroplasty by Morbidity Measure

**Morbidity scale**	**Category**	THA (N)	PYR	Incidence /100pyr	95% CI
Charlson Comorbidity Index	0	7878	64676	12.2	(11.9,12.4)
	1	1,160	11,729	9.9	(9.3,10.5)
	2	1,115	10,096	11.0	(10.4,11.7)
	3+	795	8,628	9.2	(8.6,9.9)
Count of chronic diseases	0	4,248	34,744	12.2	(11.9,12.6)
	1	3,631	30,613	11.9	(11.5,12.2)
	2	1,955	17,482	11.2	(10.7,11.7)
	3+	1,114	12,289	9.1	(8.5, 9.6)
Count of medications prescribed	0-4	4,674	38,337	12.2	(11.8,12.5)
	5-7	2,911	24,489	11.9	(11.5,12.3)
	8-12	2,421	22,576	10.7	(10.3,11.2)
	13+	942	9,726	9.7	(9.1,10.3)
Count of contacts with primary care	0-7	4,946	42,662	11.6	(11.3,11.9)
	8-11	2,559	21,483	11.9	(11.5,12.4)
	12-17	1,939	16,902	11.5	(11.0,12.0)
	18+	1,504	14,080	10.7	(10.1,11.2)
Electronic Frailty Index	0-4	8,373	69,541	12.0	(11.8,12.3)
	5-8	2,249	21,349	10.5	(10.1,11.0)
	9-12	303	3,839	7.9	(7.0,8.8)
	13+	23	399	5.8	(3.4,8.1)

For peer review only

Table 4: THA surgery rates by Morbidity Status

**Morbidity scale**	**Category**	Total at risk	Competing events	THA events	Unadjusted HR (95% CI)	Adjusted HR* (95% CI)
Charlson Comorbidity Index	0	18,303	2,279	7,878	Ref	Ref
	1	3,202	630	1,160	0.79 (0.75-0.84)	0.84 (0.79-0.89)
	2	3,261	596	1,115	0.78 (0.74-0.84)	0.81 (0.76-0.87)
	3+	2,794	656	795	0.63 (0.58-0.68)	0.65 (0.60-0.70)
Count of chronic diseases	0	9,228	1,142	4,248	Ref	Ref
	1	8,802	1,231	3,631	0.90 (0.86-0.94)	0.91 (0.87-0.95)
	2	5,339	881	1,955	0.80 (0.76-0.84)	0.81 (0.77-0.86)
	3+	4,191	907	1,114	0.57 (0.53-0.61)	0.59 (0.55-0.63)
Count of medications	0-4	10,250	1,206	4,674	Ref	Ref
	5-7	7,234	1,111	2,911	0.89 (0.85-0.93)	0.92 (0.88-0.97)
	8-12	6,857	1,169	2,421	0.78 (0.74-0.82)	0.81 (0.77-0.86)
	13+	3,219	675	942	0.64 (0.59-0.68)	0.67 (0.62-0.72)
Count of contacts with primary care	0-7	11,790	1,570	4,946	Ref	Ref
	8-11	6,272	866	2,559	1.00 (0.95-1.04)	1.02 (0.97-1.07)
	12-17	5,097	825	1,939	0.92 (0.88-0.97)	0.95 (0.91-1.01)
	18+	4,401	900	1,504	0.82 (0.77-0.87)	0.85 (0.81-0.91)
Electronic Frailty Index	0-4	19,102	2,596	8,373	Ref	Ref
	5-8	6,900	1,157	2,249	0.75 (0.71-0.78)	0.77 (0.74-0.81)
	9-12	1,391	354	303	0.48 (0.43-0.54)	0.52 (0.47-0.59)
	13+	167	54	23	0.31 (0.20-0.46)	0.34 (0.22-0.51)

For peer review only

Figure 1. Flowchart of participants

For peer review only

For peer review only

Figure: Flow chart of participants

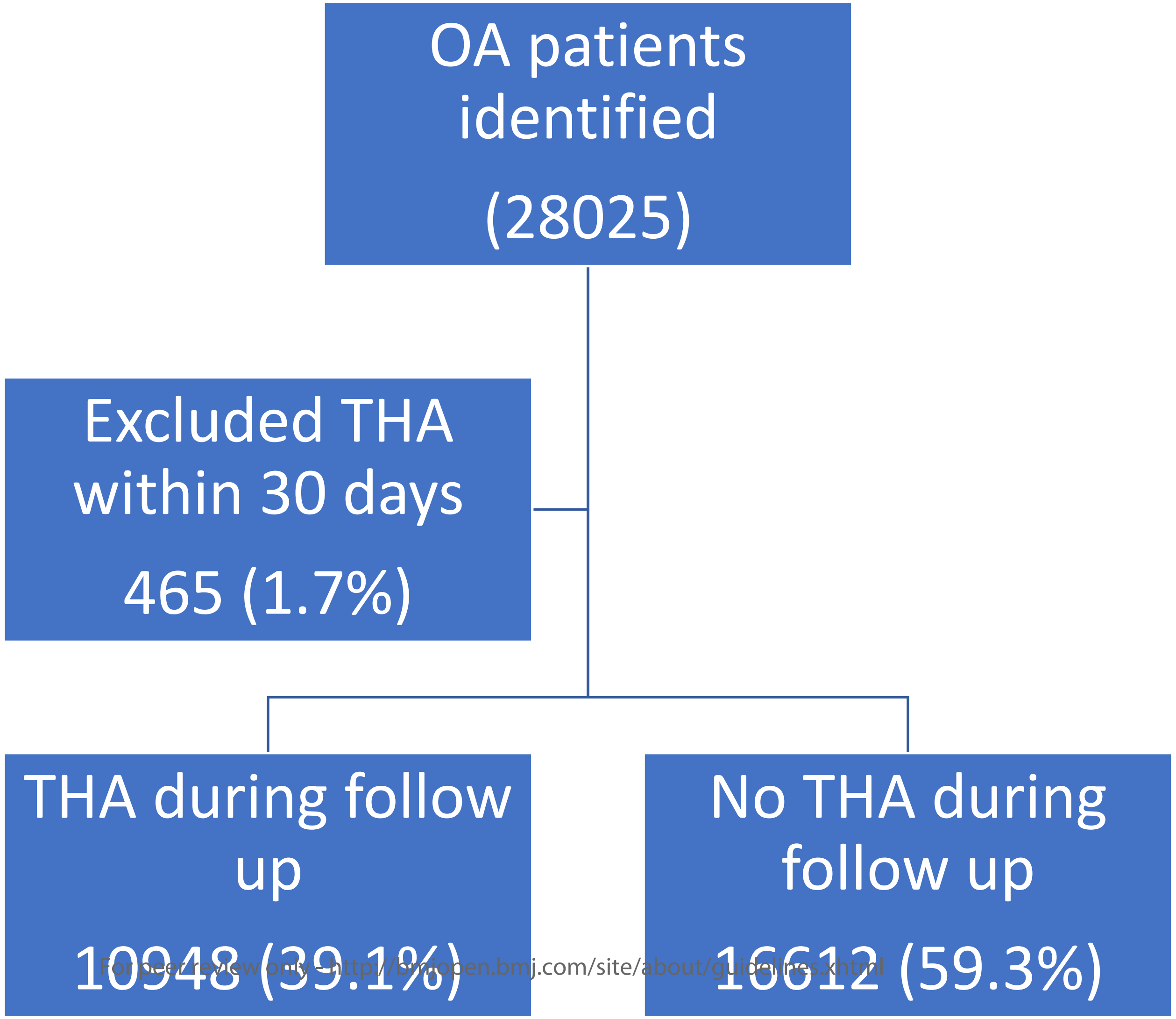

For peer review only - http://bmjopen.bmj.com/site/about/guidelines.xhtml

Supplementary File 1: Read codes for hip OA

1. N053512 Hip OA NOS
2. N05z511 Hip OA NOS
3. N05zJ00 OA NOS, of hip
4. Nyu2100 [X]Other primary coxarthrosis
5. N051900 Primary coxarthrosis, bilateral

For peer review only

Supplementary File 2: Disorders covered by the Quality and Outcomes Framework (QOF).

1. asthma
2. atrial fibrillation
3. hypertension
4. cancer
5. chronic kidney disease
6. COPD
7. Dementia
8. Depression
9. diabetes,
10. epilepsy
11. heart failure
12. learning disability
13. mental health disorder
14. osteoporosis
15. peripheral arterial disease
16. rheumatoid arthritis
17. stroke and TIA

For peer review only

For peer review only

Reporting checklist for cohort study.

Based on the STROBE cohort guidelines.

Instructions to authors

Complete this checklist by entering the page numbers from your manuscript where readers will find each of the items listed below.

Your article may not currently address all the items on the checklist. Please modify your text to include the missing information. If you are certain that an item does not apply, please write "n/a" and provide a short explanation.

Upload your completed checklist as an extra file when you submit to a journal.

In your methods section, say that you used the STROBE cohortreporting guidelines, and cite them as:

von Elm E, Altman DG, Egger M, Pocock SJ, Gotzsche PC, Vandenbroucke JP. The Strengthening the Reporting of Observational Studies in Epidemiology (STROBE) Statement: guidelines for reporting observational studies.

		Reporting Item	Page Number
**Title and abstract**			
Title	#1a	Indicate the study’s design with a commonly used term in the title or the abstract	1
Abstract	#1b	Provide in the abstract an informative and balanced summary of what was done and what was found	2
**Introduction**			
Background / rationale	#2	Explain the scientific background and rationale for the investigation being reported	4
Objectives	#3	State specific objectives, including any prespecified hypotheses	4
**Methods**			
Study design	#4	Present key elements of study design early in the paper	4
Setting	#5	Describe the setting, locations, and relevant dates, including periods of recruitment, exposure, follow-up, and data collection	4
Eligibility criteria	#6a	Give the eligibility criteria, and the sources and methods of selection of participants. Describe methods of follow-up.	4
Eligibility criteria	#6b	For matched studies, give matching criteria and number of exposed and unexposed	NA
Variables	#7	Clearly define all outcomes, exposures, predictors, potential confounders, and effect modifiers. Give diagnostic criteria, if applicable	4,5

For peer review only

Data sources / measurement	#8	For each variable of interest give sources of data and details of methods of assessment (measurement). Describe comparability of assessment methods if there is more than one group. Give information separately for for exposed and unexposed groups if applicable.	5
Bias	#9	Describe any efforts to address potential sources of bias	5,6
Study size	#10	Explain how the study size was arrived at	6
Quantitative variables	#11	Explain how quantitative variables were handled in the analyses. If applicable, describe which groupings were chosen, and why	5
Statistical methods	#12a	Describe all statistical methods, including those used to control for confounding	5,6
Statistical methods	#12b	Describe any methods used to examine subgroups and interactions	NA
Statistical methods	#12c	Explain how missing data were addressed	6
Statistical methods	#12d	If applicable, explain how loss to follow-up was addressed	NA
Statistical methods	#12e	Describe any sensitivity analyses	6
**Results**			
Participants	#13a	Report numbers of individuals at each stage of study—eg numbers potentially eligible, examined for eligibility, confirmed eligible, included in the study, completing follow-up, and analysed. Give information separately for for exposed and unexposed groups if applicable.	7
Participants	#13b	Give reasons for non-participation at each stage	NA
Participants	#13c	Consider use of a flow diagram	17
Descriptive data	#14a	Give characteristics of study participants (eg demographic, clinical, social) and information on exposures and potential confounders. Give information separately for exposed and unexposed groups if applicable.	13
Descriptive data	#14b	Indicate number of participants with missing data for each variable of interest	13
Descriptive data	#14c	Summarise follow-up time (eg, average and total amount)	7
Outcome data	#15	Report numbers of outcome events or summary measures over time. Give information separately for exposed and unexposed groups if applicable.	15,16
Main results	#16a	Give unadjusted estimates and, if applicable, confounder-adjusted estimates and their precision (eg, 95% confidence interval). Make clear which	15,16

For peer review only

		confounders were adjusted for and why they were included	
Main results	#16b	Report category boundaries when continuous variables were categorized	15,16
Main results	#16c	If relevant, consider translating estimates of relative risk into absolute risk for a meaningful time period	NA
Other analyses	#17	Report other analyses done—e.g., analyses of subgroups and interactions, and sensitivity analyses	9
**Discussion**			
Key results	#18	Summarise key results with reference to study objectives	8
Limitations	#19	Discuss limitations of the study, taking into account sources of potential bias or imprecision. Discuss both direction and magnitude of any potential bias.	8,9
Interpretation	#20	Give a cautious overall interpretation considering objectives, limitations, multiplicity of analyses, results from similar studies, and other relevant evidence.	8,9
Generalisability	#21	Discuss the generalisability (external validity) of the study results	9
**Other Information**			
Funding	#22	Give the source of funding and the role of the funders for the present study and, if applicable, for the original study on which the present article is based	10

The STROBE checklist is distributed under the terms of the Creative Commons Attribution License CC-BY. This checklist was completed on 20. October 2020 using https://www.goodreports.org/, a tool made by the EQUATOR Network in collaboration with Penelope.ai

For peer review only

BMJ Open

Influence of pre-existing multimorbidity on receiving a hip arthroplasty: Cohort study of 28,026 elderly subjects from UK primary care

Journal:	*BMJ Open*
Manuscript ID	bmjopen-2020-046713.R1
Article Type:	Original research
Date Submitted by the Author:	22-Mar-2021
Complete List of Authors:	Ferguson, Rory; University of Oxford, NDORMS<br>Prieto-Alhambra, Daniel; University of Oxford, Centre for Statistics in Medicine, NDORMS<br>Peat, George; Keele University, Research Institute for Primary Care & Health Sciences<br>Delmestri, Antonella; University of Oxford, Nuffield Department of Orthopaedics, Rheumatology, and Musculoskeletal Sciences<br>Jordan, Kelvin; Keele University, School of Primary, Community and Social Care<br>Strauss, Vicky; University of Oxford, Centre for Statistics in Medicine, Ndorms<br>Valderas, Jose; University of Exeter Medical School,<br>Walker, Christine; Keele University, Research User Group, Arthritis Research UK Primary Care Centre, Research Institute of Primary Care and Health Sciences<br>Yu, Dahai; Keele University, Research Institute for Primary Care & Health Sciences<br>Glyn-Jones, Sion; University of Oxford<br>Silman, Alan; University of Oxford Nuffield Department of Orthopaedics Rheumatology and Musculoskeletal Sciences, Botnar Institute
**Primary Subject Heading**:	Rheumatology
Secondary Subject Heading:	Geriatric medicine
Keywords:	Hip < ORTHOPAEDIC & TRAUMA SURGERY, Health policy < HEALTH SERVICES ADMINISTRATION & MANAGEMENT, SURGERY, GERIATRIC MEDICINE

SCHOLARONE™
Manuscripts

I, the Submitting Author has the right to grant and does grant on behalf of all authors of the Work (as defined in the below author licence), an exclusive licence and/or a non-exclusive licence for contributions from authors who are: i) UK Crown employees; ii) where BMJ has agreed a CC-BY licence shall apply, and/or iii) in accordance with the terms applicable for US Federal Government officers or employees acting as part of their official duties; on a worldwide, perpetual, irrevocable, royalty-free basis to BMJ Publishing Group Ltd ("BMJ") its licensees and where the relevant Journal is co-owned by BMJ to the co-owners of the Journal, to publish the Work in this journal and any other BMJ products and to exploit all rights, as set out in our licence.

The Submitting Author accepts and understands that any supply made under these terms is made by BMJ to the Submitting Author unless you are acting as an employee on behalf of your employer or a postgraduate student of an affiliated institution which is paying any applicable article publishing charge ("APC") for Open Access articles. Where the Submitting Author wishes to make the Work available on an Open Access basis (and intends to pay the relevant APC), the terms of reuse of such Open Access shall be governed by a Creative Commons licence – details of these licences and which Creative Commons licence will apply to this Work are set out in our licence referred to above.

Other than as permitted in any relevant BMJ Author's Self Archiving Policies, I confirm this Work has not been accepted for publication elsewhere, is not being considered for publication elsewhere and does not duplicate material already published. I confirm all authors consent to publication of this Work and authorise the granting of this licence.

For peer review only

Influence of pre-existing multimorbidity on receiving a hip arthroplasty: Cohort study of 28,026 elderly subjects from UK primary care

RJ Ferguson [1]
D Prieto-Alhambra [1]
G Peat [2]
A Delmestri [1]
KP Jordan [2]
V Strauss [1]
JM Valderas [3]
C Walker [2]
D Yu[2]
S Glyn-Jones [1]
AJ Silman[1,4]

[1]Nuffield Department of Orthopaedics, Rheumatology and Musculoskeletal Sciences, University of Oxford, Oxford, UK.
[2]Primary Care and Health Sciences Department, Keele University, Keele, UK.
[3]University of Exeter Medical School, Exeter, UK.
[4]Oxford University Hospital NHS Foundation Trust, Oxford, UK.

Correspondence to Professor Silman
Nuffield Department of Orthopaedics, Rheumatology and Musculoskeletal Sciences, University of Oxford
Botnar Research Centre, Windmill Road
Headington, Oxford, OX3 7LD.
alan.silman@ndorms.ox.ac.uk

Word count: abstract 289, main paper 2741

Key words: Hip osteoarthritis, arthroplasty, multimorbidity, health care, health service

This study is based in part on data from the Clinical Practice Research Datalink obtained under licence from the UK Medicines and Healthcare products Regulatory Agency. The data is provided by patients and collected by the NHS as part of their care and support. The interpretation and conclusions contained in this study are those of the author/s alone
HES Data/ONS Data shown are Copyright © 2016 are re-used with the permission of The Health & Social Care Information Centre. All rights reserved.
The OPCS Classification of Interventions and Procedures, codes, terms and text is Crown copyright (2016) published by Health and Social Care Information Centre, also known as NHS Digital and licensed under the Open Government Licence available at www.nationalarchives.gov.uk/doc/open-government-licence/open-government-licence.htm.

For peer review only

Ethical Approval

No ethical approval was required and anonymised data were provided by as above. No individual patient consent was therefore required

Consent for publication: not applicable

Acknowledgements

We acknowledge Professor Andy Judge for advice on statistical analysis at the earliest stages of this project . We are also grateful to Dr Andrew Clegg, and Stephen Pye for guidance in the use of the electronic frailty index. Dr John Griffiths and Professor Nigel Arden, provided very helpful advice at the design stage on the challenges of anaesthetic assessment and on studying osteoarthritis from an epidemiological perspective. We acknowledge the contribution of the Research Users group at Keele for discussions on the results and their interpretation

For peer review only

Abstract

Objective

To evaluate how pre-existing levels of comorbidity, assessed using 5 different approaches, influence the likelihood and timing of receiving a total hip arthroplasty (THA) in people aged 65 and over.

Setting

A longitudinal record linkage study of a UK national sample from primary care linked to their secondary care records

Participants

All 28,025 patients, newly recorded as having hip osteoarthritis identified from the national primary care register – Clinical Practice Research Datalink (CPRD) - which allowed extraction of their background health and morbidity status using 5 different constructs: Charlson Comorbidity Index, Electronic Frailty Index (eFI) and counts of comorbidity disorders (from list of 17), prescribed medications and number of primary care visits prior to recording of osteoarthritis.

Outcome measures

The record of having received a THA as recorded in the primary care record and in a linked secondary care database: HES (hospital episode statistics).

Results

40% had THA over a median follow up of 10 months (range 1 to 17 years). All approaches to assessing comorbidity were associated with a reduced likelihood of THA, although the impact varied by approach. Even low levels of comorbidity were associated with some increased peri-operative risk.

Conclusion

Markers of pre-existing poor health, not unexpectedly, will influence decision for THA in an elderly person with end stage hip osteoarthritis. although these effects are modest for most indices of comorbidity with the exception of eFI. There is also some evidence of this selection is present even in people with moderate decrements in their health, despite the balance of benefits to risk in these individuals being positive.

(260 words)

For peer review only

Strengths and limitations

- National sample of older patients recorded in primary care with newly reported osteoarthritis of the hip
- Multi-dimensional approach to assessing their concurrent morbidity and health status
- Linkage to national hospital records to assess receipt of surgery
- Challenge using routine data sources to quantify completeness and accuracy of diagnostic and treatment records
- Not able to assess impact on mutltimorbidity on referral as opposed to surgery

For peer review only

Introduction

Total hip arthroplasty (THA) is a highly cost-effective procedure for hip osteoarthritis (OA)(1). The mean age at surgery in many OECD countries is currently around 70 (2), and consequently many patients being considered for THA will have multiple comorbidities (3). Our anecdotal experience, reinforced by the patients consulted prior to this research, was that pre-existing morbidities raise concerns about operative risk and hence deter surgery thereby limiting access to those who may otherwise benefit. Balancing risks and benefits of surgical procedures, for examples invasive cardiac procedures, has been well discussed for such potentially life threatening disorders (4). There is however very little evidence to guide health care professionals on the risks and benefits of THA in elderly patients with multimorbidity but who have severe limitation of quality of life (5-6). In one report recently published from a tertiary care centre from the USA, background comorbidity was linked to both an increased risk of complications and a poorer outcome (7). The concern, though, is that those with relatively modest background levels of multimorbidity may be excluded from surgery. Our objective was to determine, using a national primary care database, how levels of pre-existing multimorbidity influence the likelihood and timing of receiving THA.

Methods

Design

In brief we undertook a cohort study analysing routinely collected data gathered from the UK's Clinical Practice Research Datalink (CPRD) (ISAC approval number 17_024R) (8). This longitudinal primary care record of elderly (patients aged 65 and over) was interrogated to identify patients with a first recorded diagnosis of hip OA. From these individuals, data from their CPRD record was used to extract information on their general health and disease status from which 5 approaches to scoring their comorbidity were derived. Patients were then followed up to identify those recorded as receiving a THA.

Subjects

These were 28,025 patients who had been diagnosed with hip OA in CPRD GOLD (see Appendix 1) records between January 1st 1995 and April 30th 2014. The diagnosis of hip OA was based on being recorded with at least one from a list of relevant READ codes (Appendix 2) (9). A validation exercise, obtaining clinical and radiographic data on a small subsample of 119 of these patients. Based on these detailed data, 88% were confirmed as having hip osteoarthritis which showed a sufficiently high level of diagnostic accuracy against accepted clinical criteria (10).

Multimorbidity markers

Information extracted from the longitudinal primary care records was used to score patients into different categories of 5 separate approaches to assessing their background morbidity and health at the time of recording of hip OA. The spectrum of measures aimed to cover (i)

For peer review only

specific risk scores accepted as predictive for post-surgical morbidity, (ii) more generalised scores of health and function and (iii) scores of health care utilisation. The specific scores used were:

1) Simple count of ever mention of the 17 chronic disorders included in the Quality and Outcomes Framework (QOF) (11) (Appendix 3)– a scheme to ensure maximal primary care compliance with recording chronic disorders
2) Charlson Comorbidity Index (CCI) based on the cumulative burden of its components over the previous 5 years. The CCI is a well-accepted measure of peri-and post-operative morbidity (12)
3) Electronic Frailty Index (eFI), a validated tool that aims to score frailty based on the number of different 'deficits' mentioned in the primary care record across a large number of health domains (13).
4) Count of the number of different individual medications in the previous 12 months via CPRD GOLD prescription data
5) Count of consultation events with primary care in the previous 12 months as a measure of health services burden

Follow up

Patients were then followed up using their CPRD record to identify those where there was a record of receiving THA based on relevant codes (14) by January 2015. As recording of THA may not be complete in CPRD, we also considered the alternative source ascertainment of THA based on linkage of CPRD records to the anonymized data from the national Hospital Episode Statistic (HES) dataset. HES linkage is only available for England not all general practices in CPRD permitted linkage. We therefore conducted a sensitivity analysis (see below) using the HES data.

Analysis

The analysis was directed towards assessing the relationship between the different categories of each of the multimorbidity scoring systems. Thus, we assessed both the cumulative incidence and the time to event of receiving a THA following the diagnosis of hip OA in the primary care record. There was a concern that in some cases the diagnostic label of hip OA may have been applied by the primary care physician once the decision to refer for surgery had been made. Thus an arbitrary decision was made to exclude those who had surgery within 30 days from the diagnosis date as coded in the record. For each of the five approaches to scoring morbidity, patients were divided into 4 categories of increasing severity. The overall incidence of THA per 100 patient years (PYR) was calculated for each category. The association between multimorbidity categories and the likelihood of THA was then investigated using Fine and Gray competing risk analysis models (15) with death being considered a competing event. The models are presented as morbidity-specific hazard ratios (HRs) with 95% confidence intervals (CIs). The lowest multimorbidity category was defined as the referent category. Univariate and then multivariate models- adjusted for age,

sex, region of the UK and calendar year of diagnosis, were conducted. We addressed the potential bias of the incompleteness of THA in the CPRD record. To assess this we undertook a sensitivity analysis in the sub set of practices with HES linkage using that latte as a source of THA

Patient and Public involvement

The research question was informed by our patient representatives both in Oxford and Keele, who helped shape the objectives and the outcome measures. One of the representatives, (author CW) contributed to the planning of the analysis. The Research Users group at Keele engaged in discussions about the results, their interpretation and dissemination. As this was a database study, there was not patient involvement in recruitment or data collection

Results

In all 28,025 patients aged over 65 had been diagnosed with hip OA in their CPRD GOLD records between January 1st 1995 and April 30th 2014. Their mean age was 75 and 62% were female. The details of their demographic characteristics are shown in Table 1.

The distributions of morbidity data using the allocation of categories for the patients, at the time of first recording of their hip OA, are shown in Table 2. The CCI scores showed that two thirds had a score of 0, a similar proportion to those who had one or less other chronic disorder. The proportion of subjects in each category of increasing score, showed broadly similar trends for the three 'count' approaches of assessing morbidity. The frailty scores based on the eFI showed that only around 6 percent could be categorised as being 'moderately' or 'severely' frail.

On follow up (Figure 1) 11,413 subjects had a THR at some stage. As discussed above, we excluded 465 who had surgery within 30 days from the diagnosis date. Of the remainder, 10,948 (39.7%) received a THA during the follow up period with a median interval of just under 10 months, range 1 month to 17 years. The overall incidence of THA during the period of observation was 11.5/100 person years, ie just over 10% per year. The THA rates by morbidity category are shown in Table 3. In this crude analysis, there was variation in the relationship between the scores of the different measures of morbidity on whether or not a patient had surgery during follow up. All the measures apart from the Charlson score showed a trend of decreasing surgery rates with increasing morbidity: the trend was steeper with eFI and only modest with the other measures. There was no trend of increasing surgery with the Charlson score.

Analysis of time to surgery, allowing for deaths during follow up are shown as hazard ratios in Table 4, both unadjusted and then adjusted for age, gender, region and year of diagnosis.

For peer review only

The cumulative incidence of surgery over time is also displayed graphically for each of the measures of morbidity in Supplementary Figures 1-5. These results show a marked trend of decreasing likelihood of surgery with increasing levels of morbidity, however defined. The graphs show that the decreased likelihood of surgery is concentrated in the earliest period of follow up. Although the decreased occurrence of surgery was not unexpected, the reduction in surgery rates was observed even in those with the least increase in their morbidity status above 'baseline'. Thus, for example, those who were only 'mildly' frail or had a CCI of 1 were 23% and 16% less likely respectively to have surgery. Adjustment for age and gender made little difference to the results suggesting that, it was the morbidity and not age that explains the data.

Discussion

This paper provides a snapshot of the levels of background morbidity and health utilisation on large national cohort of elderly patients newly presenting to their general practitioner with hip OA. Not surprisingly given concerns about operative risk, the results showed that the greater the pre-existing morbidity the longer the time to surgery. However, this influence on time to surgery is observed even in those with a low surgical risk, albeit at a minor level. In this analysis those who were just mildly frail had almost a quarter lower rate of surgery. Whether comorbidity should influence decision for elective hip surgery has been debated given the relative lack of influence on long term mortality (16). Further, in a companion paper examining the influence of comorbidity on the outcome of THA in the same UK primary care derived population, the benefits of surgery in terms of quality of life These benefits outweighed a modest increased mortality risk were similar independent of baseline morbidity (17 submitted for publication).

Strengths and limitations

The population studied was derived from a national population base, CPRD GOLD, and provided, a representative sample of patients in primary care with OA of the hip (8). A primary care data set is also the only option for providing information on the levels of morbidity at the time of recording the hip diagnosis. These could then be analysed prospectively to evaluate the relationship between morbidity and the likelihood of surgery. Comorbidity is a concept with no single measure that is perfect (18). We used a broad range of measures, each identifying a different concept of comorbidity that might have influenced surgical decision making.

There are a number of limitations that must be considered before interpreting the results further.

These data are based on the primary care recording of OA of the hip in this electronic dataset. There were likely to be errors both in terms of under-reporting of all cases and diagnostic inaccuracy in those who were included. We (DY, GP and KJ) have previously reported on a retrospective analysis of the presence of codes for hip OA specifically, and OA

For peer review only

in general, in patients who had a THA recorded in CPRD (19). In that analysis there was substantial under-reporting of hip OA when relying on a specific code of hip OA, although the under-recording was lower when allowing the primary care to mention OA in any joint. The latter would have been too non-specific for the current analysis. By contrast (as mentioned above) the level of diagnostic inaccuracy in those who were recorded as having hip OA, based on a detailed clinical review of a sample of cases was low (10). We also have no data on laterality so we had to assume that the side of the recorded OA was the same side as the surgery.

The major aim of the analysis was to study the delay (time to), rather than the fact of, surgery following the recording of the diagnosis of hip OA. Patients attending their primary care physician with hip pain or other relevant symptom may have only been recorded as having OA once a decision to refer for surgery had been made. Indeed, given the potential for under-reporting of OA of the hip, it is perhaps more likely that the OA label may have been made on those with more definite and severe OA.

These points illustrate the challenges in relying on administrative databases from primary care to identify a complete population of cases. Thus, our results on the relationship between comorbidity and THA can only refer to those with a hip OA code.

We are unable to separate from our data set the contribution that baseline comorbidity made between the decision by *primary care to refer for an orthopaedic opinion* from that made in *secondary care as to whether to have surgery*. Although in practice such a separation may be hard to interpret as the referring primary care physician may influence both the patient and the orthopaedic surgeon in their decision. In theory referral data are captured in UK primary care, but in practice this was missing for most of the patients. We are unable to address from these data at what stage and what were the predominant influences that lead to a greater reluctance for surgery.

Any study such as this reflects the decisions made in relation to factors operating in the UK health system at that point in time. Views about the need for surgery and secular changes in resource availability might have influenced our results. We therefore adjusted for year of diagnosis which made no difference. However, it is interesting to see for example how the recent CoVid-19 epidemic might influence surgical decision making currently. Indeed, the CCI is being modified to collect such information (20).

Comorbidity as assessed by our scores is not static. We examined the influence of the scores at the time of first recording of hip OA. Apart from the health care utilisation type scores (drug counts and primary care visits), the scores were based on accumulation of comorbidities (over 5 years for CCI and ever for eFI and disease count) to the time of

For peer review only

diagnosis. Given the median time between primary care recording and surgery was under a year, we suggest that these scores are unlikely to have changed to any important extent

The crude incidence rates of surgery, although showing (apart from the Charlson score) a decreasing trend with increasing level of morbidity, these trends were more pronounced when time to surgery was considered, after allowing for the greater death rates in those at greatest risk. As the survival curves suggest there was no evidence that surgery was delayed in those who had surgery and these data are shown in Supplementary Table 1.

We used CPRD GOLD as our source of data on THA which covers surgery in primary care NHS. In the NHS, data are also available from Hospital Episode Statistics (HES) which can be linked to CPRD. However, HES does not cover Scottish, Northern Ireland or Welsh patients and also linkage to HES was not available for all practices; a link was only possible for just under 17,000 of our cohorts. We did undertake a sensitivity analysis (Supplementary Table 2) using that subset. Although the total numbers were lower, the morbidity distributions were similar as were the relationships with the likelihood of surgery to the whole dataset.

Finally, there are key confounders, which might explain the results. Thus, both BMI and smoking may be key contributors to the decision as to whether a patient with OA has surgery. CPRD GOLD records are incomplete in regard to these variables and there is also concern of over adjustment. BMI might be the major predictor of the likelihood of surgery and should be examined. BMI data were available in 22,344 (79.8%) of the cohort but at varying times in relation to the record of OA. Given the lack of data of BMI at the time of osteoarthritis diagnosis it was not considered appropriate to adjust for this in the subset with a BMI record. As a check of the impact of excluding BMI as a confounder, we found only a very modest relationship between BMI category and surgery (data not shown) and did not alter the associations with morbidity in that subset.

Although, we have demonstrated in a companion paper that co-existing morbidity did not constrain clinical benefit (17) this does not capture all the reasons why surgery does not occur. Patients supported by their health care professionals may judge that their other health issues are so predominant as to increase the reluctance for surgery despite the possible benefit. When we presented the results of this study to our patient partners, this was not their expressed view, but this requires further investigation.

In conclusion modest background morbidity does influence the timing of who has THA in the UK, despite evidence that the benefits in such groups outweigh the harms

For peer review only

Contributorship Statement:
The study was conceived by AJS with SG-J and DP-A and detailed protocol produced by these authors with input from GP, KPJ, JMV and DY. AD was responsible for extracting, preparing and ensuring the quality of the datasets for analysis; analysis was undertaken by RJF supported by VS, DP-A and AJS, CW provided patient input into the design, approach to analysis and interpretation of the data. All authors contributed to the discussion of the results. The manuscript was prepared by AJS and RJF and reviewed by all authors

Competing interests:
The authors declare that they have no conflicts of interests to report

Funding
This report is independent research funded by the National Institute for Health Research (Research for Patient Benefit programme, PB-PG-0815-20024). The views expressed in this publication are those of the authors and not necessarily those of the NIHR or the Department of Health and Social Care

Data Statement: The data that support the findings of this study are available from CPRD but restrictions apply to the availability of these data, which were used under license for the current study, and so are not publicly available. The datasets extracted from CPRD and used in the analysis during the current study are available from the corresponding author on reasonable request and consistent with the permission received from CPRD

For peer review only

References

1. NHS Digital. Provisional Quarterly Patient Reported Outcome Measures (PROMs) in England. 2017. https://digital.nhs.uk/catalogue/PUB23060.
2. National Joint Registry. National Joint Registry for England, Wales, Northern Ireland and the Isle of Man: 17th Annual Report. 2020 https://reports.njrcentre.org.uk/downloads.
3. The Academy of Medical Sciences. Multimorbidity: a priority for global health research. 2018 https://acmedsci.ac.uk/file-download/82222577 (accessed April 4, 2019).
4. Orkaby AR, Forman DE. Assessing Risks and Benefits of Invasive Cardiac Procedures in Patients with Advanced Multimorbidity. *Clin Geriatr Med*. 2016;32(2):359-371. doi:10.1016/j.cger.2016.01.004
5. Gordon M, Paulsen A, Overgaard S, Garellick G, Pedersen AB, Rolfson O. Factors influencing health-related quality of life after total hip replacement--a comparison of data from the Swedish and Danish hip arthroplasty registers. *BMC Musculoskelet Disord*. 2013;14:316. Published 2013 Nov 6. doi:10.1186/1471-2474-14-316
6. Greene ME, Rolfson O, Gordon M, Garellick G, Nemes S. Standard Comorbidity Measures Do Not Predict Patient-reported Outcomes 1 Year After Total Hip Arthroplasty. *Clin Orthop Relat Res*. 2015;473(11):3370-3379. doi:10.1007/s11999-015-4195-z
7. Mannion AF, Nauer S, Arsoy D, Impellizzeri FM, Leuning M.The Association Between Comorbidity and the Risks and Early Benefits of Total Hip Arthroplasty for Hip Osteoarthritis J Arthroplasy 2020 (in press) doi.org/10.1016/j.arth.2020.04.090
8. Herrett E, Gallagher AM, Bhaskaran K, *et al.* Data Resource Profile: Clinical Practice Research Datalink (CPRD). *Int J Epidemiol* 2015; **44**: 827–36.
9. https://digital.nhs.uk/services/terminology-and-classifications/read-codes
10. Ferguson RJ, Prieto-Alhambra D, Walker C, et al. Validation of hip osteoarthritis diagnosis recording in the UK Clinical Practice Research Datalink. *Pharmacoepidemiol Drug Saf*. 2019;28(2):187-193. doi:10.1002/pds.4673
11. NHS Primary Care Commissioning *QOF implementation business rules: v11*. http://www.primarycarecontracting.nhs.uk/145.php
12. Charlson ME, Pompei P, Ales KL, MacKenzie CR. A new method of classifying prognostic comorbidity in longitudinal studies: development and validation. *J Chronic Dis* 1987; **40**: 373–83
13. Clegg A, Bates C, Young J, *et al.* Development and validation of an electronic frailty index using routine primary care electronic health record data. *Age Ageing* 2016; **45**: 353–60.
14. Royal College of Surgeons of England, Academy of Medical Royal Colleges. Hospital Episode Statistics and revalidation.2013, RCS England
15. Fine JP, Gray RJ. A proportional hazards model for the subdistribution of a competing risk. J Am Stat Assoc. 1999;94:496-509.
16. Bülow E, Rolfson O, Cnudde P et al. Comorbidity does not predict long-term mortality after total hip arthroplasty. *Acta Orthop*. 2017;88(5):472-477.
17. Fergsuon RJ, Prieto-Alhambra D, Peat G et al. Influence of pre-existing multimorbidity on receiving a hip arthroplasty: cohort study of 28,026 elderly subjects from UK primary care (submitted for publication)

18. Mehta HB, Dimou F, Adhikari D, et al. Comparison of Comorbidity Scores in Predicting:10.1097/MLR.0000000000000465
19. Yu D, Jordan KP, Peat G. Under-recording of osteoarthritis in United Kingdom primary care electronic health record data. *Clin Epidemiol*. 2018;10:1195-1201. Published 2018 Sep 12. doi:10.2147/CLEP.S160059
20. https://www.mdcalc.com/charlson-comorbidity-index-cci

For peer review only

Table 1: Demographic Characteristics of Cohort

**Variable**	**Category**	**N (28025)**	**%**
**Age**	65-69	7200	25.7
	70-74	7229	25.8
	75-79	6421	22.9
	80-84	4233	15.1
	85-89	2161	7.7
**Gender**	Male	10,662	38.0
	Female	17,363	62.0
**BMI***	<18.5	257	1.2
	18.5-<25	6,611	29.6
	25-<30	9,569	42.8
	30-<35	4,269	19.1
	35+	1,638	7.3
**IMD quintiles**	Least deprived	4,668	25.9
	2	4,503	25.0
	3	3,981	22.1
	4	3,190	17.7
	Most deprived	1,696	9.4
*no record of BMI in 5681<br>**IMD missing in 9987 as only available on ONS perfect matches and only in England			

For peer review only

1
2 Table 2 Background Morbidity Status of Cohort
3

**Morbidity scale**	**Category**	**N** 28,025	**%**
Charlson Comorbidity Index	0	18,631	66.5
	1	3,245	11.6
	2	3,312	11.8
	3+	2,837	10.1
Count of chronic diseases	0	9,398	33.5
	1	8,957	32.0
	2	5,414	19.3
	3+	4,256	15.2
Count of medications prescribed	0-4	10,392	37.1
	5-7	7,365	26.3
	8-12	6,990	24.9
	13+	3,278	11.7
Count of contacts with primary care	0-7	11,945	42.6
	8-11	6,378	22.8
	12-17	5,212	18.6
	18+	4,490	16.0
Electronic Frailty Index	0-4	19,441	69.4
	5-8	7,005	25.0
	9-12	1,411	5.0
	13+	168	0.6

4
5

For peer review only

1 Table 3. Crude Incidence of Total Hip Arthroplasty by Morbidity Measure

2

**Morbidity scale**	**Category**	THA (N)	PYR	Incidence /100pyr	95% CI
Charlson Comorbidity Index	0	7878	64676	12.2	(11.9,12.4)
	1	1,160	11,729	9.9	(9.3,10.5)
	2	1,115	10,096	11.0	(10.4,11.7)
	3+	795	8,628	9.2	(8.6,9.9)
Count of chronic diseases	0	4,248	34,744	12.2	(11.9,12.6)
	1	3,631	30,613	11.9	(11.5,12.2)
	2	1,955	17,482	11.2	(10.7,11.7)
	3+	1,114	12,289	9.1	(8.5, 9.6)
Count of medications prescribed	0-4	4,674	38,337	12.2	(11.8,12.5)
	5-7	2,911	24,489	11.9	(11.5,12.3)
	8-12	2,421	22,576	10.7	(10.3,11.2)
	13+	942	9,726	9.7	(9.1,10.3)
Count of contacts with primary care	0-7	4,946	42,662	11.6	(11.3,11.9)
	8-11	2,559	21,483	11.9	(11.5,12.4)
	12-17	1,939	16,902	11.5	(11.0,12.0)
	18+	1,504	14,080	10.7	(10.1,11.2)
Electronic Frailty Index	0-4	8,373	69,541	12.0	(11.8,12.3)
	5-8	2,249	21,349	10.5	(10.1,11.0)
	9-12	303	3,839	7.9	(7.0,8.8)
	13+	23	399	5.8	(3.4,8.1)

3

For peer review only

1 Table 4: Total Hip Arthroplasty rates by Morbidity Status

**Morbidity scale**	**Category**	Total at risk	Competing events	THR events	Unadjusted HR (95% CI)	Adjusted HR* (95% CI)
Charlson Comorbidity Index	0	18,303	2,279	7,878	Ref	Ref
	1	3,202	630	1,160	0.79 (0.75-0.84)	0.84 (0.79-0.89)
	2	3,261	596	1,115	0.78 (0.74-0.84)	0.81 (0.76-0.87)
	3+	2,794	656	795	0.63 (0.58-0.68)	0.65 (0.60-0.70)
Count of chronic diseases	0	9,228	1,142	4,248	Ref	Ref
	1	8,802	1,231	3,631	0.90 (0.86-0.94)	0.91 (0.87-0.95)
	2	5,339	881	1,955	0.80 (0.76-0.84)	0.81 (0.77-0.86)
	3+	4,191	907	1,114	0.57 (0.53-0.61)	0.59 (0.55-0.63)
Count of medications	0-4	10,250	1,206	4,674	Ref	Ref
	5-7	7,234	1,111	2,911	0.89 (0.85-0.93)	0.92 (0.88-0.97)
	8-12	6,857	1,169	2,421	0.78 (0.74-0.82)	0.81 (0.77-0.86)
	13+	3,219	675	942	0.64 (0.59-0.68)	0.67 (0.62-0.72)
Count of contacts with primary care	0-7	11,790	1,570	4,946	Ref	Ref
	8-11	6,272	866	2,559	1.00 (0.95-1.04)	1.02 (0.97-1.07)
	12-17	5,097	825	1,939	0.92 (0.88-0.97)	0.95 (0.91-1.01)
	18+	4,401	900	1,504	0.82 (0.77-0.87)	0.85 (0.81-0.91)
Electronic Frailty Index	0-4	19,102	2,596	8,373	Ref	Ref
	5-8	6,900	1,157	2,249	0.75 (0.71-0.78)	0.77 (0.74-0.81)
	9-12	1,391	354	303	0.48 (0.43-0.54)	0.52 (0.47-0.59)
	13+	167	54	23	0.31 (0.20-0.46)	0.34 (0.22-0.51)

3 *age, gender, region and year of diagnosis

For peer review only

Figure 1. Flowchart of participants (see attached file)

For peer review only

Figure: Flow chart of participants

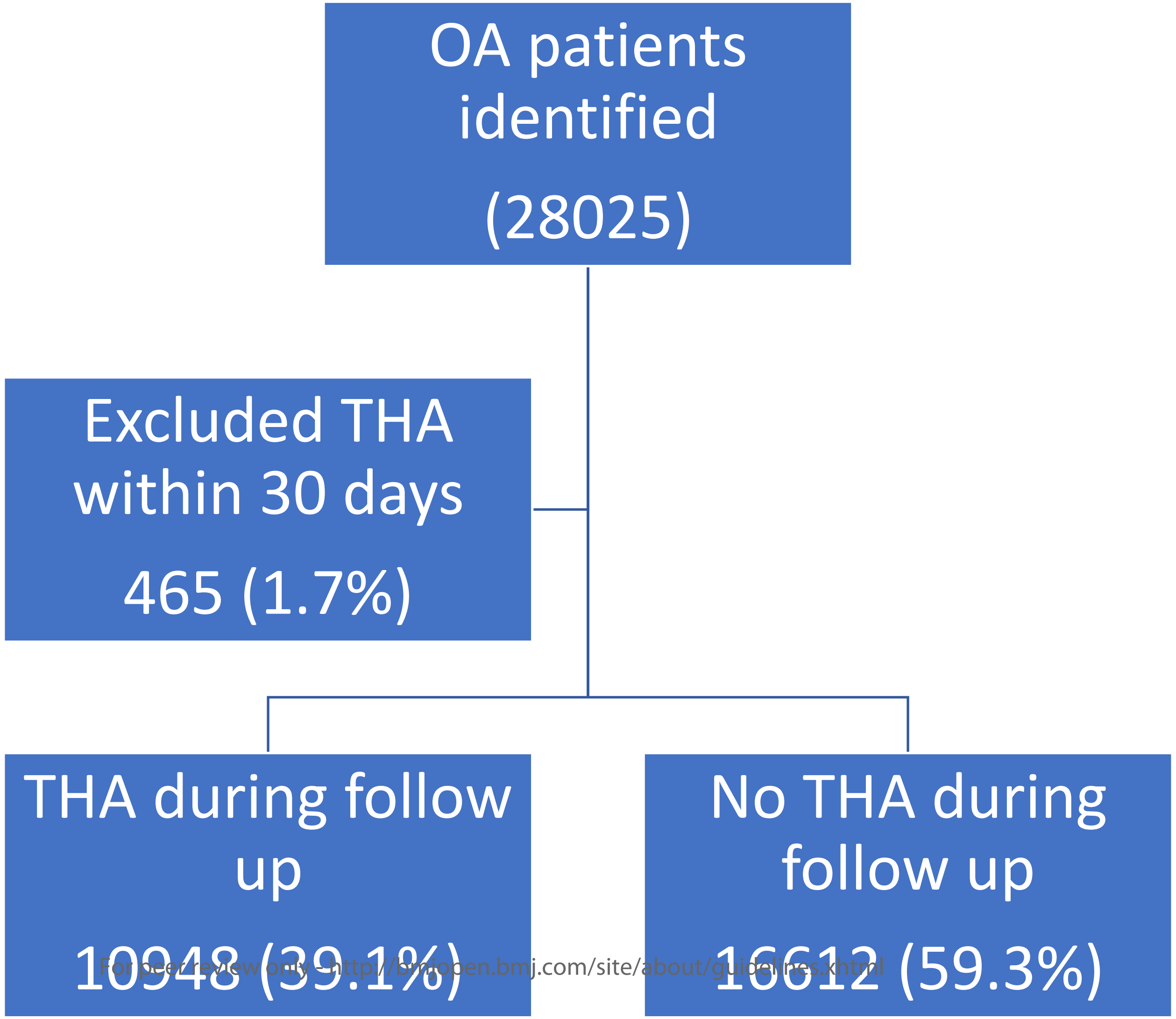

For peer review only - http://bmjopen.bmj.com/site/about/guidelines.xhtml

Appendix 1: CPRD GOLD Coverage

The CPRD was established in 1987 and is a not-for-profit research service funded by the NHS National Institute for Health Research (NIHR) and the Medicines and Healthcare products Regulatory Agency (MHRA). It is now owned by the UK Department of Health. CPRD collects data from GP practices using Vision systems, with approximately 718 in total. The nomenclature of the CPRD has evolved over time: originally termed the General Practice Research Database (GPRD), until it was launched as the CPRD in 2012. It is now termed CPRD GOLD, to differentiate it from a recently launched 'sister' database CPRD Aurum that collects data from GP practices using EMIS Web software. Henceforth, use of the term CPRD refers to CPRD GOLD. CPRD contains the records of 17 million patients, of which 4.4 million are active, covering approximately 7% of the UK population (1). Importantly, CPRD patients are broadly representative of the UK population in terms of age, sex, ethnicity and body mass index distribution (2).

1. Clinical Practice Research Datalink. https://www.cprd.com/intro.asp.

2. Herrett E, Gallagher AM, Bhaskaran K, et al. Data Resource Profile: Clinical Practice Research Datalink (CPRD). *Int J Epidemiol*. 2015;44(3):827-836. doi:10.1093/ije/dyv098

For peer review only

Appendix 2: Read codes for hip OA

1. N053512 Hip OA NOS
2. N05z511 Hip OA NOS
3. N05zJ00 OA NOS, of hip
4. Nyu2100 [X]Other primary coxarthrosis
5. N051900 Primary coxarthrosis, bilateral

For peer review only

Appendix 3: Disorders covered by the Quality and Outcomes Framework (QOF).

1. asthma
2. atrial fibrillation
3. hypertension
4. cancer
5. chronic kidney disease
6. COPD
7. Dementia
8. Depression
9. diabetes,
10. epilepsy
11. heart failure
12. learning disability
13. mental health disorder
14. osteoporosis
15. peripheral arterial disease
16. rheumatoid arthritis
17. stroke and TIA

For peer review only

Supplementary Table 1: Mean time to total hip arthroplasty by morbidity category

	Category	Mean time to surgery (years)
Charlson Comorbidity Index	0	1.46
	1	1.52
	2	1.19
	3+	1.10
Count of chronic diseases	0	1.57
	1	1.40
	2	1.28
	3+	1.10
Count of medications prescribed	0-4	1.60
	5-7	1.36
	8-12	1.21
	13+	1.17
Count of contacts with primary care	0-7	1.52
	8-11	1.40
	12-17	1.31
	18+	1.25
Electronic Frailty Index	0-4	1.49
	5-8	1.18
	9-12	0.99
	13+	1.12

For peer review only

Supplementary Table 2: Total hip arthroplasty rates by morbidity status based on HES recorded surgery

**Morbidity scale**	**Category**	Total at risk	Competing events	THR events	Unadjusted HR (95% CI)	Adjusted HR* (95% CI)
Charlson Comorbidity Index	0	10,980	1,992	4,552	Ref	Ref
	1	2,037	584	703	0.79 (0.73-0.86)	0.85 (0.78-0.92)
	2	2,034	466	707	0.86 (0.79-0.93)	0.85 (0.78-0.92)
	3+	1,732	495	525	0.72 (0.65-0.78)	0.68 (0.62-0.75)
Count of chronic diseases	0	5,559	1,059	2,412	Ref	Ref
	1	5,280	1,069	2,150	0.97 (0.91-1.03)	0.93 (0.88-0.98)
	2	3,306	724	1,182	0.86 (0.80-0.92)	0.81 (0.76-0.87)
	3+	2,638	685	743	0.68 (0.63-0.74)	0.63 (0.58-0.69)
Count of medications	0-4	6,192	1,138	2,695	Ref	Ref
	5-7	4,445	941	1,759	0.94 (0.89-1.00)	0.94 (0.89-1.00)
	8-12	4,188	943	1,474	0.85 (0.80-0.90)	0.83 (0.78-0.89)
	13+	1,958	515	559	0.70 (0.64-0.76)	0.66 (0.60-0.73)
Count of contacts with primary care	0-7	7,004	1,389	2,896	Ref	Ref
	8-11	3,817	734	1,473	0.96 (0.90-1.02)	0.95 (0.89-1.01)
	12-17	3,194	684	1,186	0.93 (0.87-0.99)	0.92 (0.85-0.98)
	18+	2,968	730	932	0.84 (0.78-0.90)	0.83 (0.77-0.89)
Electronic Frailty Index	0-4	11,318	2,239	4,793	Ref	Ref
	5-8	4,426	986	1,468	0.82 (0.77-0.87)	0.78 (0.73-0.83)
	9-12	920	266	209	0.55 (0.48-0.64)	0.54 (0.47-0.63)
	13+	119	46	17	0.35 (0.22-0.57)	0.34 (0.21-0.55)
**Total**		16,783	3,537	6,487		

For peer review only

Supplementary Figure 1: Cumulative Incidence of THR, by Charlson Comorbidity Index

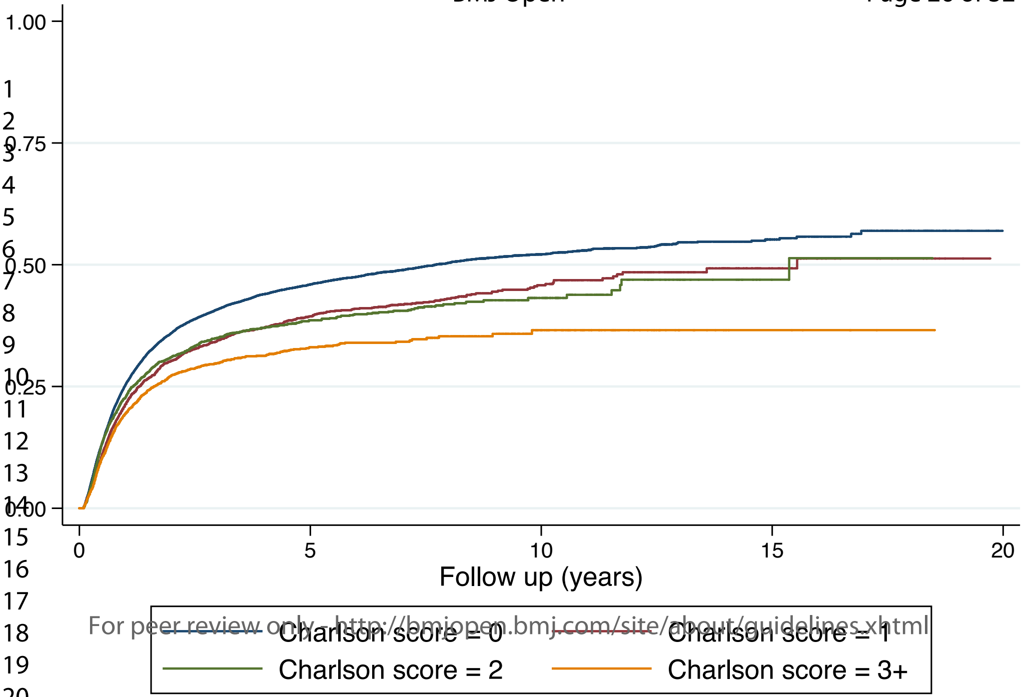

For peer review only - http://bmjopen.bmj.com/site/about/guidelines.xhtml

Supplementary Figure 2: Cumulative Incidence of THR, by count of chronic diseases

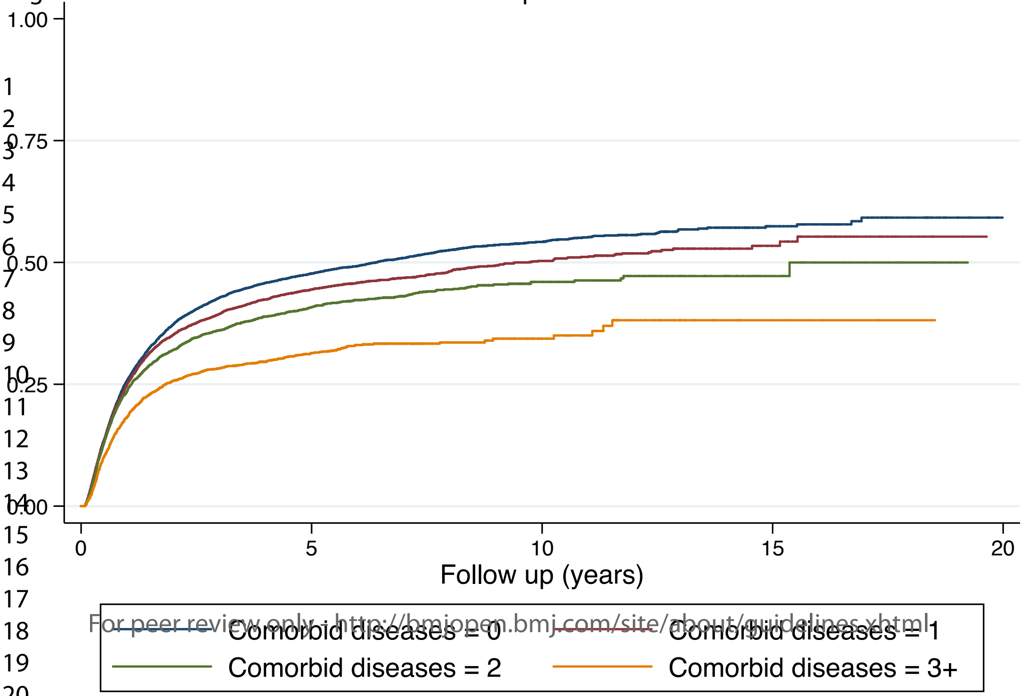

For peer review only - http://bmjopen.bmj.com/site/about/guidelines.xhtml

Supplementary Figure 3: Cumulative Incidence of THR, by count of medications

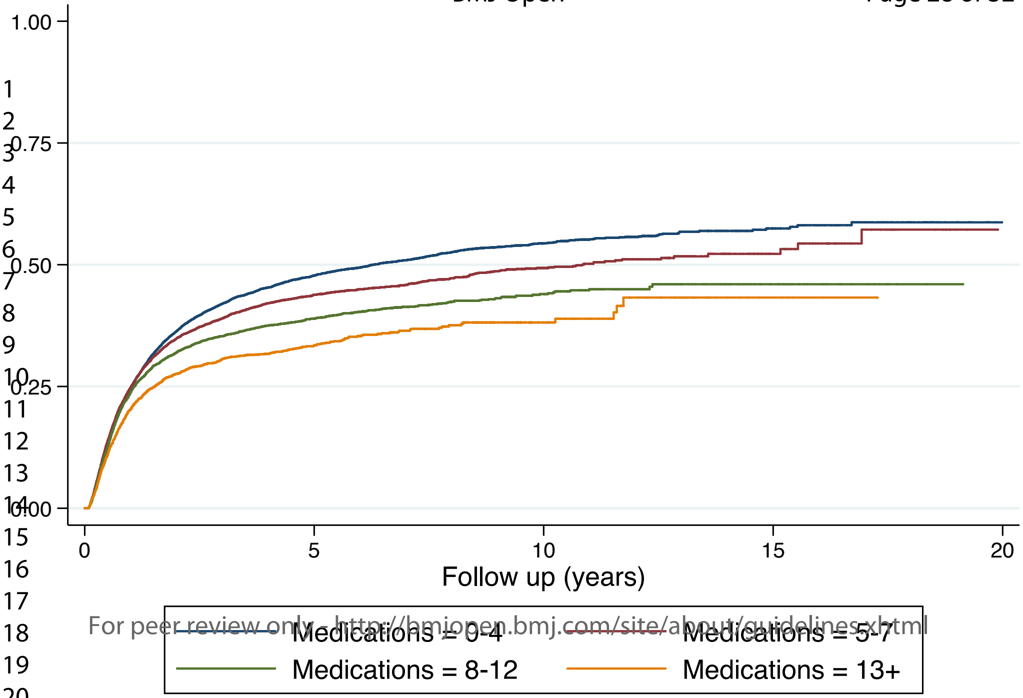

Supplementary Figure 4: Cumulative Incidence of THR, by count of primary care contacts

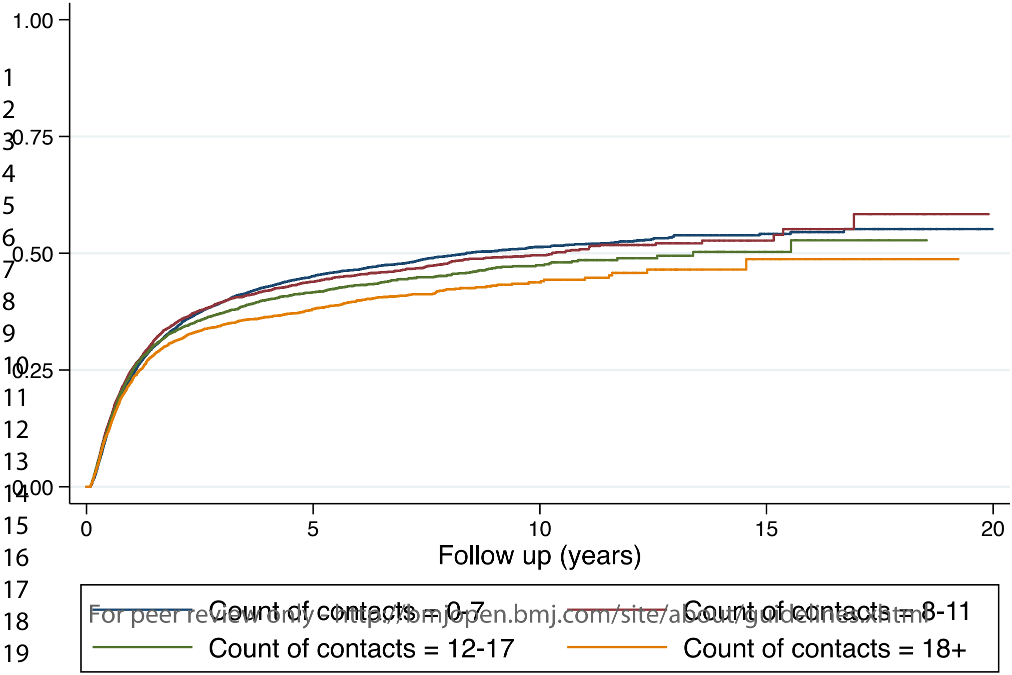

For peer review only - http://bmjopen.bmj.com/site/about/guidelines.xhtml

Supplementary Figure 5: Cumulative Incidence of THR, by Electronic Frailty Index

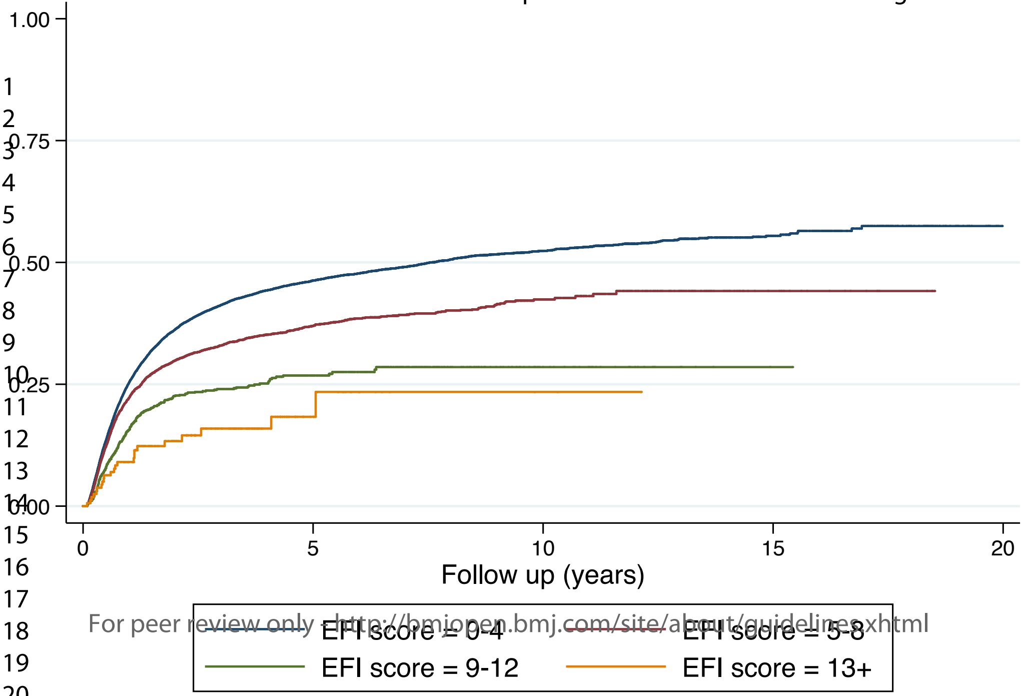

For peer review only - http://bmjopen.bmj.com/site/about/guidelines.xhtml

Reporting checklist for cohort study.

Based on the STROBE cohort guidelines.

Instructions to authors

Complete this checklist by entering the page numbers from your manuscript where readers will find each of the items listed below.

Your article may not currently address all the items on the checklist. Please modify your text to include the missing information. If you are certain that an item does not apply, please write "n/a" and provide a short explanation.

Upload your completed checklist as an extra file when you submit to a journal.

In your methods section, say that you used the STROBE cohort reporting guidelines, and cite them as:

von Elm E, Altman DG, Egger M, Pocock SJ, Gotzsche PC, Vandenbroucke JP. The Strengthening the Reporting of Observational Studies in Epidemiology (STROBE) Statement: guidelines for reporting observational studies.

		Reporting Item	Page Number (lines)
**Title and abstract**			
Title	#1a	Indicate the study's design with a commonly used term in the title or the abstract	1
Abstract	#1b	Provide in the abstract an informative and balanced summary of what was done and what was found	3
**Introduction**			
Background / rationale	#2	Explain the scientific background and rationale for the investigation being reported	5 (2-15
Objectives	#3	State specific objectives, including any prespecified hypotheses	5 (15-16)
**Methods**			
Study design	#4	Present key elements of study design early in the paper	5(21-27)
Setting	#5	Describe the setting, locations, and relevant dates, including periods of recruitment, exposure, follow-up, and data collection	5 (30-36)
Eligibility criteria	#6a	Give the eligibility criteria, and the sources and methods of selection of participants. Describe methods of follow-up.	5 (30-36)
Eligibility criteria	#6b	For matched studies, give matching criteria and number of exposed and unexposed	NA
Variables	#7	Clearly define all outcomes, exposures, predictors, potential confounders, and effect modifiers. Give diagnostic criteria, if applicable	5 (39) – 6 (25)

For peer review only

Data sources / measurement	#8	For each variable of interest give sources of data and details of methods of assessment (measurement). Describe comparability of assessment methods if there is more than one group. Give information separately for for exposed and unexposed groups if applicable.	5 (39) – 6 (25)
Bias	#9	Describe any efforts to address potential sources of bias	5 (33-36)
Study size	#10	Explain how the study size was arrived at	5 (30-31)
Quantitative variables	#11	Explain how quantitative variables were handled in the analyses. If applicable, describe which groupings were chosen, and why	6 (28-36)
Statistical methods	#12a	Describe all statistical methods, including those used to control for confounding	6 (36) –7 (4)
Statistical methods	#12b	Describe any methods used to examine subgroups and interactions	NA
Statistical methods	#12c	Explain how missing data were addressed	7 (1-4)
Statistical methods	#12d	If applicable, explain how loss to follow-up was addressed	NA
Statistical methods	#12e	Describe any sensitivity analyses	7 (1-4)
**Results**			
Participants	#13a	Report numbers of individuals at each stage of study—eg numbers potentially eligible, examined for eligibility, confirmed eligible, included in the study, completing follow-up, and analysed. Give information separately for for exposed and unexposed groups if applicable.	Figure
Participants	#13b	Give reasons for non-participation at each stage	NA
Participants	#13c	Consider use of a flow diagram	Figure
Descriptive data	#14a	Give characteristics of study participants (eg demographic, clinical, social) and information on exposures and potential confounders. Give information separately for exposed and unexposed groups if applicable.	13
Descriptive data	#14b	Indicate number of participants with missing data for each variable of interest	13
Descriptive data	#14c	Summarise follow-up time (eg, average and total amount)	7 (20-23)
Outcome data	#15	Report numbers of outcome events or summary measures over time. Give information separately for exposed and unexposed groups if applicable.	15,16
Main results	#16a	Give unadjusted estimates and, if applicable, confounder-adjusted estimates and their precision (eg, 95% confidence interval). Make clear which	15,16

For peer review only

		confounders were adjusted for and why they were included	
Main results	#16b	Report category boundaries when continuous variables were categorized	15,16
Main results	#16c	If relevant, consider translating estimates of relative risk into absolute risk for a meaningful time period	NA
Other analyses	#17	Report other analyses done—e.g., analyses of subgroups and interactions, and sensitivity analyses	Supp tables 1,2
**Discussion**			
Key results	#18	Summarise key results with reference to study objectives	8 (5-16)
Limitations	#19	Discuss limitations of the study, taking into account sources of potential bias or imprecision. Discuss both direction and magnitude of any potential bias.	8 (19) – 10 (27)
Interpretation	#20	Give a cautious overall interpretation considering objectives, limitations, multiplicity of analyses, results from similar studies, and other relevant evidence.	8-10
Generalisability	#21	Discuss the generalisability (external validity) of the study results	9 (9-20)
**Other Information**			
Funding	#22	Give the source of funding and the role of the funders for the present study and, if applicable, for the original study on which the present article is based	1 (46)

The STROBE checklist is distributed under the terms of the Creative Commons Attribution License CC-BY. This checklist was completed on 20. October 2020 using https://www.goodreports.org/, a tool made by the EQUATOR Network in collaboration with Penelope.ai

For peer review only

BMJ Open

Influence of pre-existing multimorbidity on receiving a hip arthroplasty: Cohort study of 28,026 elderly subjects from UK primary care

Journal:	*BMJ Open*
Manuscript ID	bmjopen-2020-046713.R2
Article Type:	Original research
Date Submitted by the Author:	09-Jun-2021
Complete List of Authors:	Ferguson, Rory; University of Oxford, NDORMS<br>Prieto-Alhambra, Daniel; University of Oxford, Centre for Statistics in Medicine, NDORMS<br>Peat, George; Keele University, Research Institute for Primary Care & Health Sciences<br>Delmestri, Antonella; University of Oxford, Nuffield Department of Orthopaedics, Rheumatology, and Musculoskeletal Sciences<br>Jordan, Kelvin; Keele University, School of Primary, Community and Social Care<br>Strauss, Vicky; University of Oxford, Centre for Statistics in Medicine, Ndorms<br>Valderas, Jose; University of Exeter Medical School,<br>Walker, Christine; Keele University, Research User Group, Arthritis Research UK Primary Care Centre, Research Institute of Primary Care and Health Sciences<br>Yu, Dahai; Keele University, Research Institute for Primary Care & Health Sciences<br>Glyn-Jones, Sion; University of Oxford<br>Silman, Alan; University of Oxford Nuffield Department of Orthopaedics Rheumatology and Musculoskeletal Sciences, Botnar Institute
**Primary Subject Heading**:	Rheumatology
Secondary Subject Heading:	Geriatric medicine
Keywords:	Hip < ORTHOPAEDIC & TRAUMA SURGERY, Health policy < HEALTH SERVICES ADMINISTRATION & MANAGEMENT, SURGERY, GERIATRIC MEDICINE

SCHOLARONE™
Manuscripts

BMJ

I, the Submitting Author has the right to grant and does grant on behalf of all authors of the Work (as defined in the below author licence), an exclusive licence and/or a non-exclusive licence for contributions from authors who are: i) UK Crown employees; ii) where BMJ has agreed a CC-BY licence shall apply, and/or iii) in accordance with the terms applicable for US Federal Government officers or employees acting as part of their official duties; on a worldwide, perpetual, irrevocable, royalty-free basis to BMJ Publishing Group Ltd ("BMJ") its licensees and where the relevant Journal is co-owned by BMJ to the co-owners of the Journal, to publish the Work in this journal and any other BMJ products and to exploit all rights, as set out in our licence.

The Submitting Author accepts and understands that any supply made under these terms is made by BMJ to the Submitting Author unless you are acting as an employee on behalf of your employer or a postgraduate student of an affiliated institution which is paying any applicable article publishing charge ("APC") for Open Access articles. Where the Submitting Author wishes to make the Work available on an Open Access basis (and intends to pay the relevant APC), the terms of reuse of such Open Access shall be governed by a Creative Commons licence – details of these licences and which Creative Commons licence will apply to this Work are set out in our licence referred to above.

Other than as permitted in any relevant BMJ Author's Self Archiving Policies, I confirm this Work has not been accepted for publication elsewhere, is not being considered for publication elsewhere and does not duplicate material already published. I confirm all authors consent to publication of this Work and authorise the granting of this licence.

For peer review only

Influence of pre-existing multimorbidity on receiving a hip arthroplasty: Cohort study of 28,026 elderly subjects from UK primary care

Rory J Ferguson [1]
Daniel Prieto-Alhambra [1]
George Peat [2]
Antonella Delmestri [1]
Kelvin P Jordan [2]
Victoria Strauss [1]
Jose M Valderas [3]
Christine Walker [2]
Dahai Yu[2]
Sion Glyn-Jones [1]
Alan J Silman[1]

[1]Nuffield Department of Orthopaedics, Rheumatology and Musculoskeletal Sciences, University of Oxford, Oxford, UK.
[2]Primary Care and Health Sciences Department, Keele University, Keele, UK.
[3]University of Exeter Medical School, Exeter, UK.

Correspondence to Professor Silman
Nuffield Department of Orthopaedics, Rheumatology and Musculoskeletal Sciences, University of Oxford
Botnar Research Centre, Windmill Road
Headington, Oxford, OX3 7LD.
alan.silman@ndorms.ox.ac.uk

Word count main paper 2313

Key words: Hip osteoarthritis, arthroplasty, multimorbidity, health care, health service

Ethical Approval
No ethical approval was required and anonymised data were provided by as above. No individual patient consent was therefore required

Consent for publication: not applicable

Acknowledgements

We acknowledge Professor Andy Judge for advice on statistical analysis at the earliest stages of this project . We are also grateful to Dr Andrew Clegg, and Stephen Pye for guidance in the use of the electronic frailty index. Dr John Griffiths and Professor Nigel Arden, provided very helpful advice at

For peer review only

the design stage on the challenges of anaesthetic assessment and on studying osteoarthritis from an epidemiological perspective. We also acknowledge the contribution of the patient members of the Research Users Group from Keele in for their input, especially in the interpretation of the results

For peer review only

Abstract

The median age for total hip arthroplasty (THA) is over 70 with the corollary that many individuals have multiple comorbidities. Despite the predicted improvement in quality of life, THA might be denied even to those with low levels of comorbidity.

Objective

To evaluate how pre-existing levels of comorbidity influence the likelihood and timing of THA.

Setting

Longitudinal record linkage study of a UK national sample linking their primary care l to their secondary care records

Participants

All 28,025 patients, newly recorded as having hip osteoarthritis identified from the national primary care register – Clinical Practice Research Datalinks (CPRD) which allowed extraction of their background health and morbidity status using 5 different constructs: Charlson Comorbidity Index, Electronic Frailty Index (eFI)and counts of comorbidity disorders (from list of 17), prescribed medications and number of primary care visits prior to recording of osteoarthritis.

Outcome measures

The record of having received a THA as recorded in the primary care record and the linked secondary care database: HES (hospital episode statistics).

Results

40% had THA: median follow 10 months (range 1 to 17 years). All approaches to assessing comorbidity were associated with a reduced THA likelihood, although the impact varied by approach. Using a survival analysis, compared to those in the lowest stratum of health for each of these approaches, apart for the number of primary care visits, even the next lowest level of comorbidity was associated with some increased risk of a patient progressing to THA.

Conclusion

Markers of pre-existing ill-health will influence decision for THA in the elderly with end stage hip osteoarthritis; although these effects are modest for indices of comorbidity other than eFI. There is evidence of this influence being present even in people with moderate decrements in their health, despite the balance of benefits to risk in these individuals being positive.

(300 words)

For peer review only

Strengths and limitation

- National sample of older patients recorded in primary care with newly reported osteoarthritis of the hip
- Multi-dimensional approach to assessing their concurrent morbidity and health status
- Linkage to national hospital records to assess receipt of surgery
- Challenge using such routine data source to quantify completeness and accuracy of diagnostic and treatment records
- Not able to assess impact on multimorbidity on referral as opposed to surgery

For peer review only

Introduction

Total hip arthroplasty (THA) is a highly cost-effective procedure for hip osteoarthritis (OA)(1). The mean age at surgery in many OECD countries is currently around 70 (2), and consequently many patients being considered for THA will have multiple comorbidities (3). Our anecdotal experience, reinforced by the patients' consulted prior to this research, was that pre-existing morbidities raise concerns about operative risk and hence deter surgery thereby limiting access to those who may otherwise benefit. Balancing risks and benefits of surgical procedures, for examples invasive cardiac procedures, has been well discussed for such potentially life threatening disorders (4). There is however very little evidence to guide health care professionals on the risks and benefits of THA in elderly patients with multimorbidity but who have severe limitation of quality of life (5-6). In one report recently published from a tertiary care centre from the USA, background comorbidity was linked to both an increased risk of complications and a poorer outcome (7). The concern, though, is that those with relatively modest background levels of multimorbidity may be excluded from surgery. Our objective was to determine, using a national primary care database, how levels of pre-existing multimorbidity influenced the likelihood and timing of receiving THA.

Methods

Design

In brief we undertook a cohort study analysing routinely collected data gathered from the UK's Clinical Practice Research Datalinks (CPRD) (ISAC approval number 17_024R) (8). This longitudinal primary care record of elderly (patients aged 65 and over) was interrogated to identify patients with a first recorded diagnosis of hip OA. From these individuals, data from their CPRD record was used to extract information on their general health and disease status from which 5 approaches to scoring their comorbidity were derived. Patients were then followed up to identify those recorded as receiving a THA.

Subjects

These were 28,025 patients who had been diagnosed with hip OA in CPRD GOLD (see Appendix 1) records between January 1st 1995 and April 30th 2014. The diagnosis of hip OA was based on being recorded with at least one from a list of relevant READ codes (Appendix 2) (9). A validation exercise, obtaining clinical and radiographic data on a small subsample of 119 of these patients. Based on these detailed data, 88% were confirmed as having hip osteoarthritis which showed a sufficiently high level of diagnostic accuracy against accepted clinical criteria (10).

Multimorbidity markers

Information extracted from the longitudinal primary care records was used to score patients into different categories of 5 separate approaches to assessing their background morbidity and health at the time of recording of hip OA. The spectrum of measures aimed to cover (i)

For peer review only

specific risk scores accepted as predictive for post-surgical morbidity, (ii) more generalised scores of health and function and (iii) scores of health care utilisation. The specific scores used were:

1) Simple count of ever mention of the 17 chronic disorders included in the Quality and Outcomes Framework (QOF) (11) (Appendix 3)– a scheme to ensure maximal primary care compliance with recording chronic disorders
2) Charlson Comorbidity Index (CCI) based on the cumulative burden of its components over the previous 5 years. The CCI is a well-accepted measure of peri-and post-operative morbidity (12)
3) Electronic Frailty Index (eFI), a validated tool that aims to score frailty based on the number of different 'deficits' mentioned in the primary care record across a large number of health domains (13).
4) Count of the number of different individual medications in the previous 12 months via CPRD GOLD prescription data
5) Count of consultation events with primary care in the previous 12 months as a measure of health services burden

Follow up

Patients were then followed up using their CPRD record to identify those where there was a record of receiving THA based on relevant codes (14) by January 2015. As recording of THA may not be complete in CPRD, we also considered the alternative source ascertainment of THA based on linkage of CPRD records to the anonymized data from the national Hospital Episode Statistic (HES) dataset. HES linkage is only available for England not all general practices in CPRD permitted linkage. We therefore conducted a sensitivity analysis (see below) using the HES data.

Analysis

The analysis was directed towards assessing the relationship between the different categories of each of the multimorbidity scoring systems. Thus, we assessed both the cumulative incidence and the time to event of receiving a THA following the diagnosis of hip OA in the primary care record. There was a concern that in some cases the diagnostic label of hip OA may have been applied by the primary care physician once the decision to refer for surgery had been made. Thus an arbitrary decision was made to exclude those who had surgery within 30 days from the diagnosis date as coded in the record. For each of the five approaches to scoring morbidity, patients were divided into 4 categories of increasing severity. The overall incidence of THA per 100 patient years (PYR) was calculated for each category. The association between multimorbidity categories and the likelihood of THA was then investigated using Fine and Gray competing risk analysis models (15) with death being considered a competing event. The models are presented as morbidity-specific hazard ratios (HRs) with 95% confidence intervals (CIs). The lowest multimorbidity category was defined as the referent category. Univariate and then multivariate models- adjusted for age,

sex, region of the UK and calendar year of diagnosis, were conducted. We addressed the potential bias of the incompleteness of THA in the CPRD record. To assess this, we undertook a sensitivity analysis in the sub set of practices with HES linkage using that latte as a source of THA

Patient and public Involvement

This research was funded by the NIHR Research for Patient Benefit scheme which has an absolute requirement that there is patient and public involvement in all relevant stages of the research. The research question had been originally raised in a "Priority Setting Partnership for Priorities for Research in Hip and Knee Arthroplasty" and this was followed by a survey of members of the Keele PPI panel: Research Users Group (RUG). We then tested the suggested questions with the group and received very positive feedback. One of the RUG members (Author CW), herself a patient with direct experience of the target of the research, then became an active member of the research team, participated in all the meetings and advised on the design including the questions that should be asked during the analysis stage. As part of the dissemination phase, Keele PPI group organised a round table event attended by the lead authors where the results were discussed and guidance given on how those members of the public who were present wished to see disseminated

Results

In all 28,025 patients aged over 65 had been diagnosed with hip OA in their CPRD GOLD records between January 1st 1995 and April 30th 2014. Their mean age was 75 and 62% were female. The details of their demographic characteristics are shown in Table 1.

The distributions of morbidity data using the allocation of categories for the patients, at the time of first recording of their hip OA, are shown in Table 2. The CCI scores showed that two thirds had a score of 0, a similar proportion to those who had one or less other chronic disorder. The proportion of subjects in each category of increasing score, showed broadly similar trends for the three 'count' approaches of assessing morbidity. The frailty scores based on the eFI showed that only around 6 percent could be categorised as being 'moderately' or 'severely' frail.

On follow up (Figure 1)11,413 subjects had a THR at some stage. As discussed above, we excluded 465 who had surgery within 30 days from the diagnosis date. Of the remainder, 10,948 (39.7%) received a THA during the follow up period with a median interval of just under 10 months, range 1 month to 17 years. The overall incidence of THA during the period of observation was 11.5/100 person years, ie just over 10% per year. The THA rates by morbidity category are shown in Table 3. In this crude analysis, there was variation in the relationship between the scores of the different measures of morbidity on whether or not a patient had surgery during follow up. All the measures apart from the Charlson score

For peer review only - http://bmjopen.bmj.com/site/about/guidelines.xhtml

showed a trend of decreasing surgery rates with increasing morbidity: the trend was steeper with eFI and only modest with the other measures. There was no trend of increasing surgery with the Charlson score

Analysis of time to surgery, allowing for deaths during follow up are shown as hazard ratios in Table 4, both unadjusted and then adjusted for age, gender, region and year of diagnosis. The cumulative incidence of surgery over time is also displayed graphically for each of the measures of morbidity in Supplementary Figures 1-5. These results show a marked trend of decreasing likelihood of surgery with increasing levels of morbidity, however defined. The graphs show that the decreased likelihood of surgery is concentrated in the earliest period of follow up. Although the decreased occurrence of surgery was not unexpected, the reduction in surgery rates was observed even in those with the least increase in their morbidity status above 'baseline. Thus, for example, those who were only 'mildly' frail or had a CCI of 1 were 23% and 16% less likely respectively to have surgery. Adjustment for age and gender made little difference to the results suggesting that, it was the morbidity and not age that explains the data

Discussion

This paper describes provides a snapshot of the levels of background morbidity and health utilisation on large national cohort of elderly patients newly presenting to their general practitioner with hip OA. Not surprisingly given concerns about operative risk, the results showed that the greater the pre-existing morbidity the longer the time to surgery. However, this influence on time to surgery is observed even in those with a low surgical risk, albeit at a minor level. In this analysis those who were just mildly frail had almost a quarter lower rate of surgery. Whether comorbidity should influence decision for elective hip surgery has been debated given the relative lack of influence on long term mortality (16). Further, in a companion paper examining the influence of comorbidity on the outcome of THA in the same UK primary care derived population, the benefits of surgery in terms of quality of life These benefits outweighed a modest increased mortality risk were similar independent of baseline morbidity (17 submitted for publication).

Strengths and limitations

The population studied was derived from a national population base, CPRD GOLD, and provided, a representative sample of patients in primary care with OA of the hip (8). A primary care data set is also the only option for providing information on the levels of morbidity at the time of recording the hip diagnosis. These could then be analysed prospectively to evaluate the relationship between morbidity and the likelihood of surgery. Comorbidity is a concept with no single measure that is perfect (18). We used a broad range of measures, each identifying a different concept of comorbidity that might have influenced surgical decision making.

For peer review only

There are a number of limitations that must be considered before interpreting the results further.

These data are based on the primary care recording of OA of the hip in this electronic dataset. There were likely to be errors both in terms of under-reporting of all cases and diagnostic inaccuracy in those who were included. We (DY, GP and KJ) have previously reported on a retrospective analysis of the presence of codes for hip OA specifically, and OA in general, in patients who had a THA recorded in CPRD (19). In that analysis there was substantial under-reporting of hip OA when relying on a specific code of hip OA, although the under-recording was lower when allowing the primary care to mention OA in any joint. The latter would have been too non-specific for the current analysis. By contrast (as mentioned above) the level of diagnostic inaccuracy in those who were recorded as having hip OA, based on a detailed clinical review of a sample of cases was low (10). We also have no data on laterality so we had to assume that the side of the recorded OA was the same side as the surgery

The major aim of the analysis was to study the delay (time to), rather than the fact of, surgery following the recording of the diagnosis of hip OA. Patients attending their primary care physician with hip pain or other relevant symptom may have only been recorded as having OA once a decision to refer for surgery had been made. Indeed, given the potential for under-reporting of OA of the hip, it is perhaps more likely that the OA label may have been made on those with more definite and severe OA. We also made the assumption that the side of diagnosis of hip OA was the side that was operated on.

These points illustrate the challenges in relying on administrative databases from primary care to identify a complete population of cases. Thus, our results on the relationship between comorbidity and THA can only refer to those with a hip OA code.

We are unable to separate from our data set the contribution that baseline comorbidity made between the decision by *primary care to refer for an orthopaedic opinion* from that made in *secondary care as to whether to have surgery*. Although in practice such a separation may be hard to interpret as the referring primary care physician may influence both the patient and the orthopaedic surgeon in their decision. In theory referral data are captured in UK primary care, but in practice this was missing for most of the patients. We are unable to address from these data at what stage and what were the predominant influences that lead to a greater reluctance for surgery.

Any study such as this reflects the decisions made in relation to factors operating in the UK health system at that point in time. Views about the need for surgery and secular changes in resource availability might have influenced our results. We therefore adjusted for year of diagnosis which made no difference. However, it is interesting to see for example how the

For peer review only

recent CoVid-19 epidemic might influence surgical decision making currently. Indeed, the CCI is being modified to collect such information (20).

CPRD provides access to the detailed primary care record which permitted the unique opportunity to derive the comorbidity scores analysed in this study. Given the routine nature of the data gathering there is scope for errors in the accuracy of the individual components and the limitations of CPRD are well described (8). Comorbidity as assessed by our scores is not static. We examined the influence of the scores at the time of first recording of hip OA. Apart from the health care utilisation type scores (drug counts and primary care visits), the scores were based on accumulation of comorbidities (over 5 years for CCI and ever for eFI and disease count) to the time of diagnosis. Given the median time between primary care recording and surgery was under a year, we suggest that these scores are unlikely to have changed to any important extent

The crude incidence rates of surgery, although showing (apart from the Charlson score) a decreasing trend with increasing level of morbidity, these trends were more pronounced when time to surgery using a survival analysis, after allowing for the greater death rates in those at greatest risk, as well as other possible confounders including age. There is a separate question which refers to those for whom the decision for surgery had been made. That is was there any influence of health pre-operative health status on delay to surgery. This analysis, suggest if anything, that the interval between diagnosis and surgery was, if anything, slightly shorter in those with worse health. (See Supplementary Table 1) One explanation for this somewhat paradoxical result, is that once the decision had been made to have surgery in the more 'at risk groups' then there was no reason to have any additional delay

We used CPRD GOLD as our source of data on THA which covers surgery in primary care NHS. In the NHS, data are also available from Hospital Episode Statistics (HES) which can be linked to CPRD. However, HES does not cover Scottish, Northern Ireland or Welsh patients and also linkage to HES was not available for all practices; a link was only possible for just under 17,000 of our cohorts. We did undertake a sensitivity analysis (Supplementary Table 2) using that subset. Although the total numbers were lower, the morbidity distributions were similar as were the relationships with the likelihood of surgery to the whole dataset

Finally, there are key confounders, which might explain the results. Thus, both BMI and smoking may be key contributors to the decision as to whether a patient with OA has surgery. CPRD GOLD records are incomplete in regard to these variables and there is also concern of over adjustment. BMI might be the major predictor of the likelihood of surgery and should be examined. BMI data were available in 22344 (79.8%) of the cohort but at

For peer review only

varying times in relation to the record of OA. Given the lack of data of BMI at the time of osteoarthritis diagnosis it was not considered appropriate to adjust for this in the subset with a BMI record. As a check of the impact of excluding BMI as a confounder, we found only a very modest relationship between BMI category and surgery (data not shown) and did not alter the associations with morbidity in that subset.

Although, we have demonstrated in a companion paper that co-existing morbidity did not constrain clinical benefit (17) this does not capture all the reasons why surgery does not occur. Patients supported by their health care professionals may judge that their other health issues are so predominant to increase the reluctance for surgery despite the possible benefit. When we presented the results of this study to our patient partners, this was not their expressed view, but this requires further investigation.

In conclusion modest background morbidity does influence the timing of who has THA in the UK, despite evidence that the harms in such groups outweigh the benefits

Contributor Statement:
The study was conceived by AJS with SG-J and DP-A and detailed protocol produced by these authors with input from GP, KPJ, JMV and DY. AD was responsible for extracting, preparing and ensuring the quality of the datasets for analysis; analysis was undertaken by RJF supported by VS, DP-A and AJS, CW provided patient input into the design, approach to analysis and interpretation of the data. All authors contributed to the discussion of the results. The manuscript was prepared by AJS and RJF and reviewed by all authors

Competing interests:
The authors declare that they have no conflicts of interests to report

Funding
This report is independent research funded by the National Institute for Health Research (Research for Patient Benefit programme, PB-PG-0815-20024). The views expressed in this publication are those of the authors and not necessarily those of the NIHR or the Department of Health and Social Care

This study is based in part on data from the Clinical Practice Research Datalink obtained under licence from the UK Medicines and Healthcare products Regulatory Agency. The data is provided by patients and collected by the NHS as part of their care and support. The interpretation and conclusions contained in this study are those of the author/s alone
HES Data/ONS Data shown are Copyright © 2016 are re-used with the permission of The Health & Social Care Information Centre. All rights reserved.
The OPCS Classification of Interventions and Procedures, codes, terms and text is Crown copyright (2016) published by Health and Social Care Information Centre, also known as NHS Digital and licensed under the Open Government Licence available at www.nationalarchives.gov.uk/doc/open-government-licence/open-government-licence.htm.

Data sharing statement: The data that support the findings of this study are available from CPRD but restrictions apply to the availability of these data, which were used under license for the current

For peer review only

study, and so are not publicly available. The datasets extracted from CPRD and used in the analysis during the current study are available from the corresponding author on reasonable request and consistent with the permission received from CPRD

For peer review only

References

1. NHS Digital. Provisional Quarterly Patient Reported Outcome Measures (PROMs) in England. 2017. https://digital.nhs.uk/catalogue/PUB23060.
2. National Joint Registry. National Joint Registry for England, Wales, Northern Ireland and the Isle of Man: 17th Annual Report. 2020 https://reports.njrcentre.org.uk/downloads.
3. The Academy of Medical Sciences. Multimorbidity: a priority for global health research. 2018 https://acmedsci.ac.uk/file-download/82222577 (accessed April 4, 2019).
4. Orkaby AR, Forman DE. Assessing Risks and Benefits of Invasive Cardiac Procedures in Patients with Advanced Multimorbidity. *Clin Geriatr Med*. 2016;32(2):359-371. doi:10.1016/j.cger.2016.01.004
5. Gordon M, Paulsen A, Overgaard S, Garellick G, Pedersen AB, Rolfson O. Factors influencing health-related quality of life after total hip replacement--a comparison of data from the Swedish and Danish hip arthroplasty registers. *BMC Musculoskelet Disord*. 2013;14:316. Published 2013 Nov 6. doi:10.1186/1471-2474-14-316
6. Greene ME, Rolfson O, Gordon M, Garellick G, Nemes S. Standard Comorbidity Measures Do Not Predict Patient-reported Outcomes 1 Year After Total Hip Arthroplasty. *Clin Orthop Relat Res*. 2015;473(11):3370-3379. doi:10.1007/s11999-015-4195-z
7. Mannion AF, Nauer S, Arsoy D, Impellizzeri FM, Leuning M.The Association Between Comorbidity and the Risks and Early Benefits of Total Hip Arthroplasty for Hip Osteoarthritis J Arthroplasy 2020 (in press) doi.org/10.1016/j.arth.2020.04.090
8. Herrett E, Gallagher AM, Bhaskaran K, *et al.* Data Resource Profile: Clinical Practice Research Datalink (CPRD). *Int J Epidemiol* 2015; **44**: 827–36.
9. https://digital.nhs.uk/services/terminology-and-classifications/read-codes
10. Ferguson RJ, Prieto-Alhambra D, Walker C, et al. Validation of hip osteoarthritis diagnosis recording in the UK Clinical Practice Research Datalink. *Pharmacoepidemiol Drug Saf*. 2019;28(2):187-193. doi:10.1002/pds.4673
11. NHS Primary Care Commissioning *QOF implementation business rules: v11*. http://www.primarycarecontracting.nhs.uk/145.php
12. Charlson ME, Pompei P, Ales KL, MacKenzie CR. A new method of classifying prognostic comorbidity in longitudinal studies: development and validation. *J Chronic Dis* 1987; **40**: 373–83
13. Clegg A, Bates C, Young J, *et al.* Development and validation of an electronic frailty index using routine primary care electronic health record data. *Age Ageing* 2016; **45**: 353–60.
14. Royal College of Surgeons of England, Academy of Medical Royal Colleges. Hospital Episode Statistics and revalidation.2013, RCS England
15. Fine JP, Gray RJ. A proportional hazards model for the subdistribution of a competing risk. J Am Stat Assoc. 1999;94:496-509.
16. Bülow E, Rolfson O, Cnudde P et al. Comorbidity does not predict long-term mortality after total hip arthroplasty. *Acta Orthop*. 2017;88(5):472-477.
17. Fergsuon submitted
18. Mehta HB, Dimou F, Adhikari D, et al. Comparison of Comorbidity Scores in Predicting:10.1097/MLR.0000000000000465

For peer review only

19. Yu D, Jordan KP, Peat G. Under-recording of osteoarthritis in United Kingdom primary care electronic health record data. *Clin Epidemiol*. 2018;10:1195-1201. Published 2018 Sep 12. doi:10.2147/CLEP.S160059
20. https://www.mdcalc.com/charlson-comorbidity-index-cci

For peer review only

Table 1: Demographic Characteristics of Cohort

**Variable**	**Category**	**N (28025)**	**%**
**Age**	65-69	7200	25.7
	70-74	7229	25.8
	75-79	6421	22.9
	80-84	4233	15.1
	85-89	2161	7.7
**Gender**	Male	10,662	38.0
	Female	17,363	62.0
**BMI***	<18.5	257	1.2
	18.5-<25	6,611	29.6
	25-<30	9,569	42.8
	30-<35	4,269	19.1
	35+	1,638	7.3
**IMD quintiles**	Least deprived	4,668	25.9
	2	4,503	25.0
	3	3,981	22.1
	4	3,190	17.7
	Most deprived	1,696	9.4
*no record of BMI in 5681<br>**IMD missing in 9987 as only available on ONS perfect matches and only in England			

For peer review only

1
2 Table 2 Background Morbidity Status of Cohort
3

**Morbidity scale**	**Category**	**N** 28,025	**%**
Charlson Comorbidity Index	0	18,631	66.5
	1	3,245	11.6
	2	3,312	11.8
	3+	2,837	10.1
Count of chronic diseases	0	9,398	33.5
	1	8,957	32.0
	2	5,414	19.3
	3+	4,256	15.2
Count of medications prescribed	0-4	10,392	37.1
	5-7	7,365	26.3
	8-12	6,990	24.9
	13+	3,278	11.7
Count of contacts with primary care	0-7	11,945	42.6
	8-11	6,378	22.8
	12-17	5,212	18.6
	18+	4,490	16.0
Electronic Frailty Index	0-4	19,441	69.4
	5-8	7,005	25.0
	9-12	1,411	5.0
	13+	168	0.6

4
5

For peer review only

1 Table 3. Crude Incidence of Total Hip Arthroplasty by Morbidity Measure

2

**Morbidity scale**	**Category**	THA (N)	PYR	Incidence /100pyr	95% CI
Charlson Comorbidity Index	0	7878	64676	12.2	(11.9,12.4)
	1	1,160	11,729	9.9	(9.3,10.5)
	2	1,115	10,096	11.0	(10.4,11.7)
	3+	795	8,628	9.2	(8.6,9.9)
Count of chronic diseases	0	4,248	34,744	12.2	(11.9,12.6)
	1	3,631	30,613	11.9	(11.5,12.2)
	2	1,955	17,482	11.2	(10.7,11.7)
	3+	1,114	12,289	9.1	(8.5, 9.6)
Count of medications prescribed	0-4	4,674	38,337	12.2	(11.8,12.5)
	5-7	2,911	24,489	11.9	(11.5,12.3)
	8-12	2,421	22,576	10.7	(10.3,11.2)
	13+	942	9,726	9.7	(9.1,10.3)
Count of contacts with primary care	0-7	4,946	42,662	11.6	(11.3,11.9)
	8-11	2,559	21,483	11.9	(11.5,12.4)
	12-17	1,939	16,902	11.5	(11.0,12.0)
	18+	1,504	14,080	10.7	(10.1,11.2)
Electronic Frailty Index	0-4	8,373	69,541	12.0	(11.8,12.3)
	5-8	2,249	21,349	10.5	(10.1,11.0)
	9-12	303	3,839	7.9	(7.0,8.8)
	13+	23	399	5.8	(3.4,8.1)

3

For peer review only

Table 4: THA surgery rates by Morbidity Status

**Morbidity scale**	**Category**	Total at risk	Competing events	THR events	Unadjusted HR (95% CI)	Adjusted HR* (95% CI)
Charlson Comorbidity Index	0	18,303	2,279	7,878	Ref	Ref
	1	3,202	630	1,160	0.79 (0.75-0.84)	0.84 (0.79-0.89)
	2	3,261	596	1,115	0.78 (0.74-0.84)	0.81 (0.76-0.87)
	3+	2,794	656	795	0.63 (0.58-0.68)	0.65 (0.60-0.70)
Count of chronic diseases	0	9,228	1,142	4,248	Ref	Ref
	1	8,802	1,231	3,631	0.90 (0.86-0.94)	0.91 (0.87-0.95)
	2	5,339	881	1,955	0.80 (0.76-0.84)	0.81 (0.77-0.86)
	3+	4,191	907	1,114	0.57 (0.53-0.61)	0.59 (0.55-0.63)
Count of medications	0-4	10,250	1,206	4,674	Ref	Ref
	5-7	7,234	1,111	2,911	0.89 (0.85-0.93)	0.92 (0.88-0.97)
	8-12	6,857	1,169	2,421	0.78 (0.74-0.82)	0.81 (0.77-0.86)
	13+	3,219	675	942	0.64 (0.59-0.68)	0.67 (0.62-0.72)
Count of contacts with primary care	0-7	11,790	1,570	4,946	Ref	Ref
	8-11	6,272	866	2,559	1.00 (0.95-1.04)	1.02 (0.97-1.07)
	12-17	5,097	825	1,939	0.92 (0.88-0.97)	0.95 (0.91-1.01)
	18+	4,401	900	1,504	0.82 (0.77-0.87)	0.85 (0.81-0.91)
Electronic Frailty Index	0-4	19,102	2,596	8,373	Ref	Ref
	5-8	6,900	1,157	2,249	0.75 (0.71-0.78)	0.77 (0.74-0.81)
	9-12	1,391	354	303	0.48 (0.43-0.54)	0.52 (0.47-0.59)
	13+	167	54	23	0.31 (0.20-0.46)	0.34 (0.22-0.51)

*age, gender, region and year of diagnosis

For peer review only

Figure 1. Flowchart of participants (see attached file)

For peer review only

Figure: Flow chart of participants

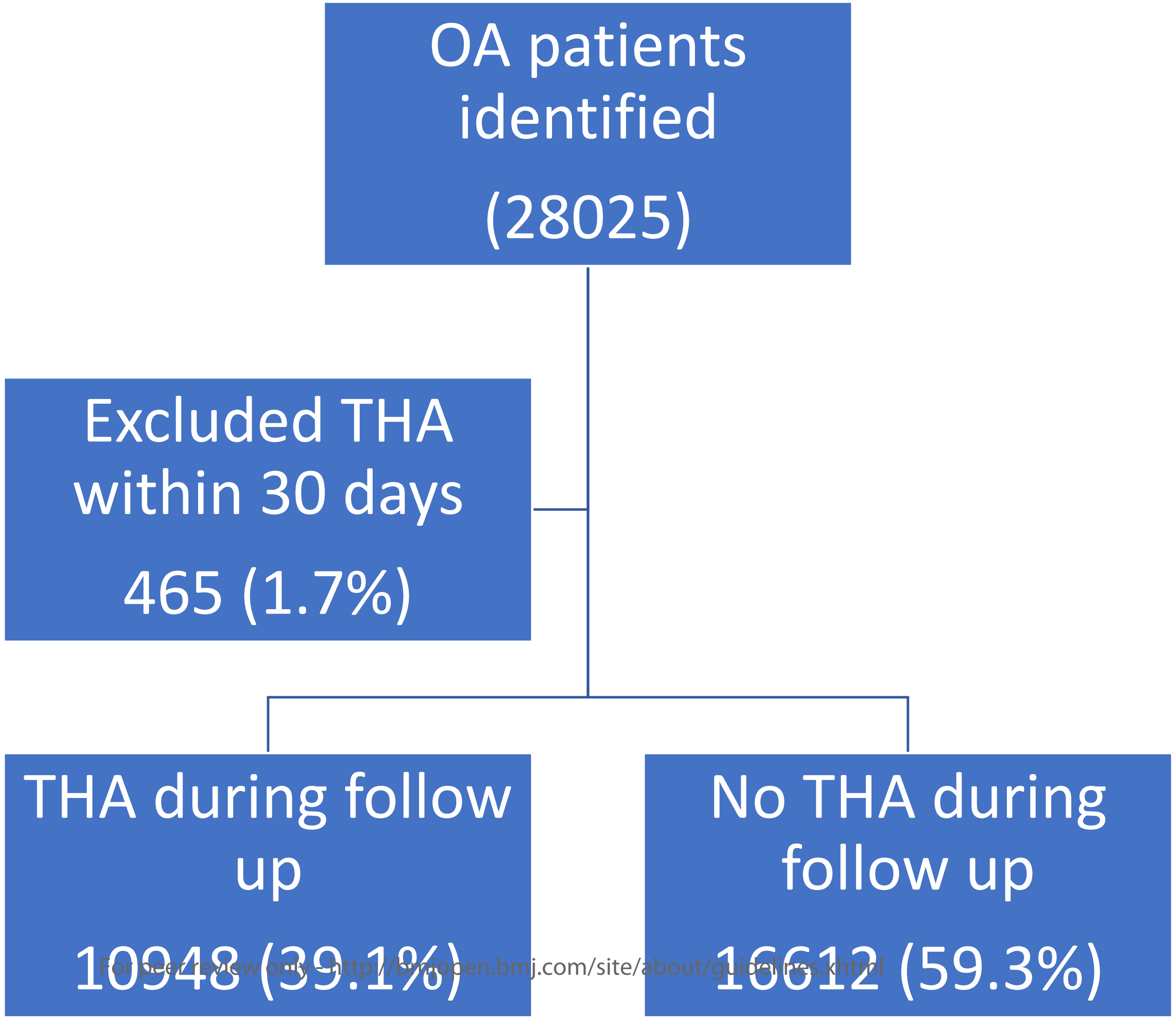

For peer review only - http://bmjopen.bmj.com/site/about/guidelines.xhtml

Appendix 1: CPRD GOLD Coverage

The CPRD was established in 1987 and is a not-for-profit research service funded by the NHS National Institute for Health Research (NIHR) and the Medicines and Healthcare products Regulatory Agency (MHRA). It is now owned by the UK Department of Health. CPRD collects data from GP practices using Vision systems, with approximately 718 in total. The nomenclature of the CPRD has evolved over time: originally termed the General Practice Research Database (GPRD), until it was launched as the CPRD in 2012. It is now termed CPRD GOLD, to differentiate it from a recently launched 'sister' database CPRD Aurum that collects data from GP practices using EMIS Web software. Henceforth, use of the term CPRD refers to CPRD GOLD. CPRD contains the records of 17 million patients, of which 4.4 million are active, covering approximately 7% of the UK population (1). Importantly, CPRD patients are broadly representative of the UK population in terms of age, sex, ethnicity and body mass index distribution (2).

1. Clinical Practice Research Datalink. https://www.cprd.com/intro.asp.

2. Herrett E, Gallagher AM, Bhaskaran K, et al. Data Resource Profile: Clinical Practice Research Datalink (CPRD). *Int J Epidemiol*. 2015;44(3):827-836. doi:10.1093/ije/dyv098

Appendix 2: Read codes for hip OA

1. N053512 Hip OA NOS
2. N05z511 Hip OA NOS
3. N05zJ00 OA NOS, of hip
4. Nyu2100 [X]Other primary coxarthrosis
5. N051900 Primary coxarthrosis, bilateral

For peer review only

Appendix 3: Disorders covered by the Quality and Outcomes Framework (QOF).

1. asthma
2. atrial fibrillation
3. hypertension
4. cancer
5. chronic kidney disease
6. COPD
7. Dementia
8. Depression
9. diabetes,
10. epilepsy
11. heart failure
12. learning disability
13. mental health disorder
14. osteoporosis
15. peripheral arterial disease
16. rheumatoid arthritis
17. stroke and TIA

For peer review only

Supplementary Table 1: Mean time to surgery by morbidity category

	Category	Mean time to surgery (years)
Charlson Comorbidity Index	0	1.46
	1	1.52
	2	1.19
	3+	1.10
Count of chronic diseases	0	1.57
	1	1.40
	2	1.28
	3+	1.10
Count of medications prescribed	0-4	1.60
	5-7	1.36
	8-12	1.21
	13+	1.17
Count of contacts with primary care	0-7	1.52
	8-11	1.40
	12-17	1.31
	18+	1.25
Electronic Frailty Index	0-4	1.49
	5-8	1.18
	9-12	0.99
	13+	1.12

For peer review only

Supplementary Table 2: THA surgery rates by Morbidity Status based on HES recorded surgery

**Morbidity scale**	**Category**	Total at risk	Competing events	THR events	Unadjusted HR (95% CI)	Adjusted HR* (95% CI)
Charlson Comorbidity Index	0	10,980	1,992	4,552	Ref	Ref
	1	2,037	584	703	0.79 (0.73-0.86)	0.85 (0.78-0.92)
	2	2,034	466	707	0.86 (0.79-0.93)	0.85 (0.78-0.92)
	3+	1,732	495	525	0.72 (0.65-0.78)	0.68 (0.62-0.75)
Count of chronic diseases	0	5,559	1,059	2,412	Ref	Ref
	1	5,280	1,069	2,150	0.97 (0.91-1.03)	0.93 (0.88-0.98)
	2	3,306	724	1,182	0.86 (0.80-0.92)	0.81 (0.76-0.87)
	3+	2,638	685	743	0.68 (0.63-0.74)	0.63 (0.58-0.69)
Count of medications	0-4	6,192	1,138	2,695	Ref	Ref
	5-7	4,445	941	1,759	0.94 (0.89-1.00)	0.94 (0.89-1.00)
	8-12	4,188	943	1,474	0.85 (0.80-0.90)	0.83 (0.78-0.89)
	13+	1,958	515	559	0.70 (0.64-0.76)	0.66 (0.60-0.73)
Count of contacts with primary care	0-7	7,004	1,389	2,896	Ref	Ref
	8-11	3,817	734	1,473	0.96 (0.90-1.02)	0.95 (0.89-1.01)
	12-17	3,194	684	1,186	0.93 (0.87-0.99)	0.92 (0.85-0.98)
	18+	2,968	730	932	0.84 (0.78-0.90)	0.83 (0.77-0.89)
Electronic Frailty Index	0-4	11,318	2,239	4,793	Ref	Ref
	5-8	4,426	986	1,468	0.82 (0.77-0.87)	0.78 (0.73-0.83)
	9-12	920	266	209	0.55 (0.48-0.64)	0.54 (0.47-0.63)
	13+	119	46	17	0.35 (0.22-0.57)	0.34 (0.21-0.55)
**Total**		16,783	3,537	6,487		

For peer review only

Supplementary Figure 1: Cumulative Incidence of THR, by Charlson Comorbidity Index

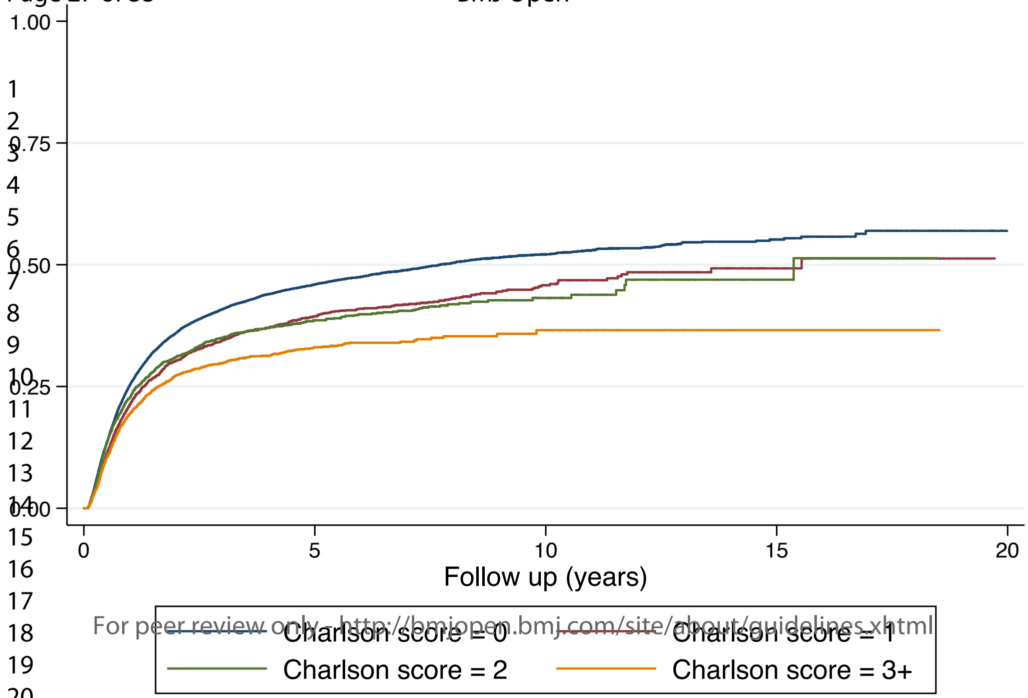

For peer review only - http://bmjopen.bmj.com/site/about/guidelines.xhtml

Supplementary Figure 2: Cumulative Incidence of THR, by count of chronic diseases

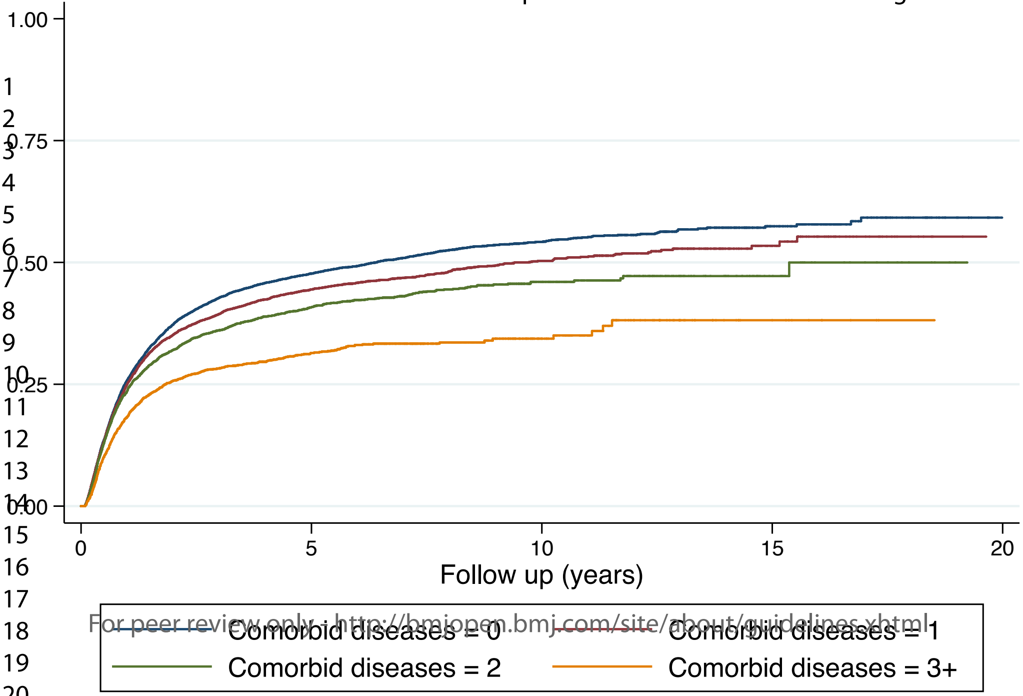

Supplementary Figure 3: Cumulative Incidence of THR, by count of medications

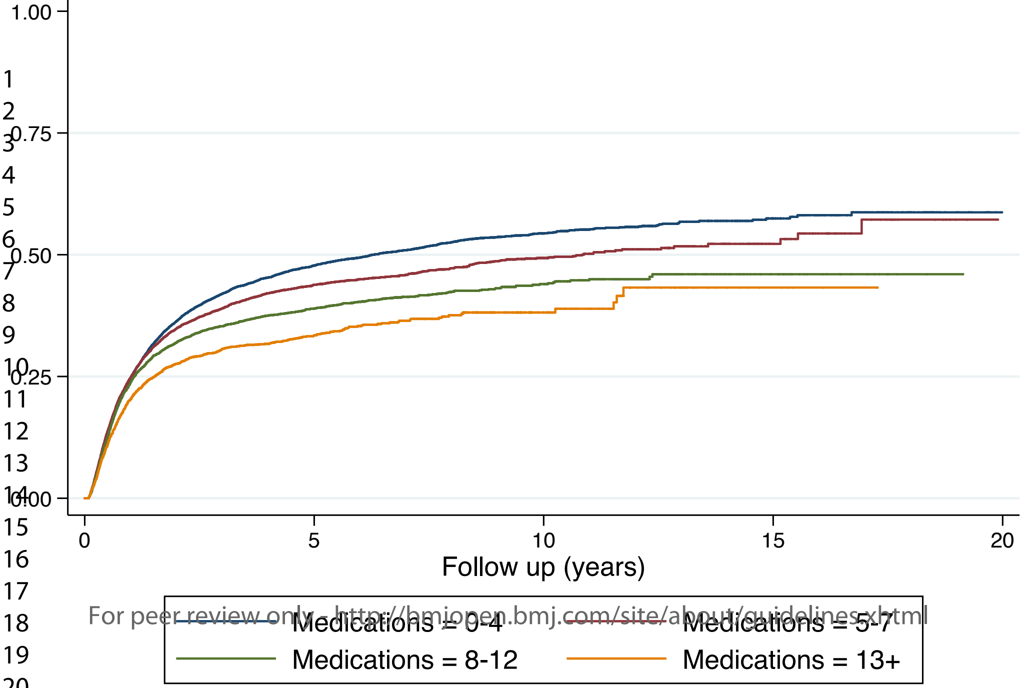

For peer review only - http://bmjopen.bmj.com/site/about/guidelines.xhtml

Supplementary Figure 4: Cumulative Incidence of THR, by count of primary care contacts

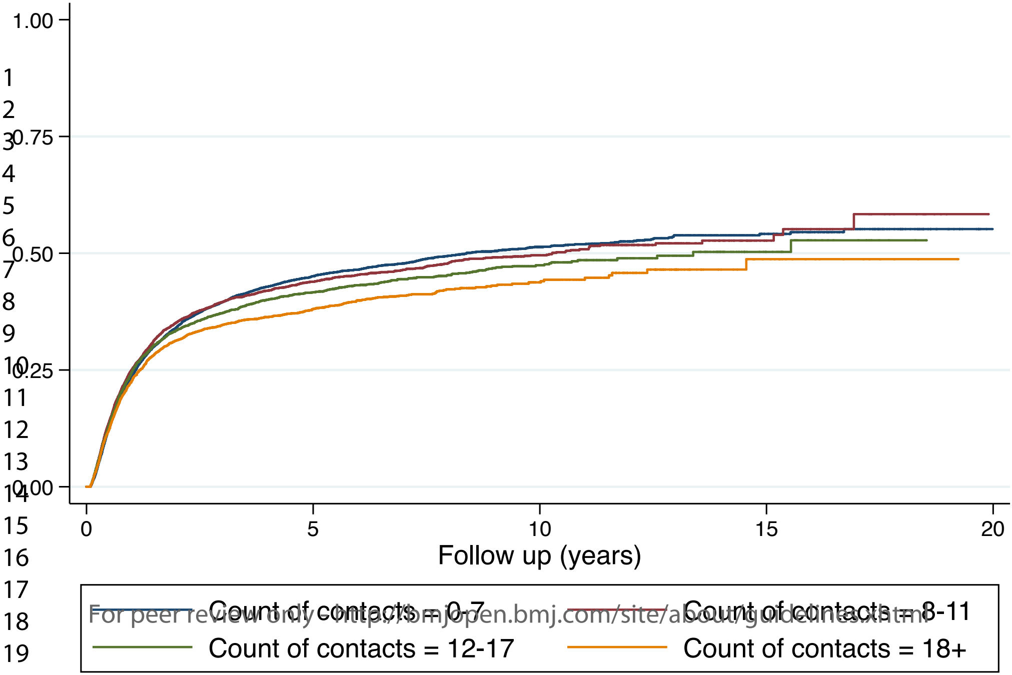

For peer review only - http://bmjopen.bmj.com/site/about/guidelines.xhtml

Supplementary Figure 5: Cumulative Incidence of THR, by Electronic Frailty Index

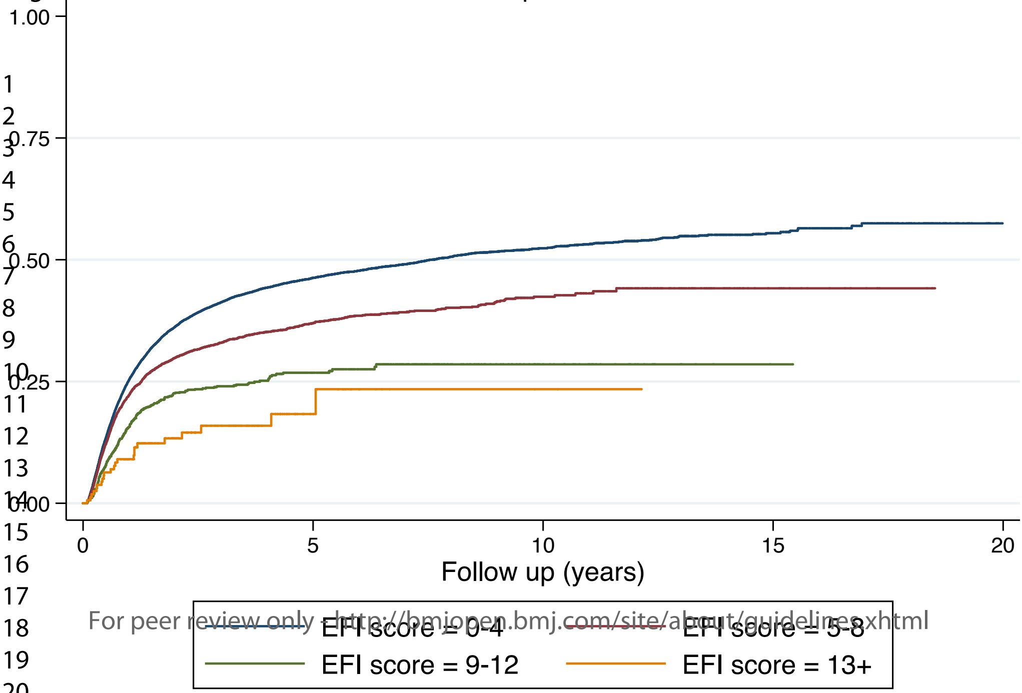

For peer review only - http://bmjopen.bmj.com/site/about/guidelines.xhtml

Reporting checklist for cohort study.

Based on the STROBE cohort guidelines.

Instructions to authors

Complete this checklist by entering the page numbers from your manuscript where readers will find each of the items listed below.

Your article may not currently address all the items on the checklist. Please modify your text to include the missing information. If you are certain that an item does not apply, please write "n/a" and provide a short explanation.

Upload your completed checklist as an extra file when you submit to a journal.

In your methods section, say that you used the STROBE cohort reporting guidelines, and cite them as:

von Elm E, Altman DG, Egger M, Pocock SJ, Gotzsche PC, Vandenbroucke JP. The Strengthening the Reporting of Observational Studies in Epidemiology (STROBE) Statement: guidelines for reporting observational studies.

		Reporting Item	Page Number (lines)
**Title and abstract**			
Title	#1a	Indicate the study's design with a commonly used term in the title or the abstract	1
Abstract	#1b	Provide in the abstract an informative and balanced summary of what was done and what was found	3
**Introduction**			
Background / rationale	#2	Explain the scientific background and rationale for the investigation being reported	5 (2-15
Objectives	#3	State specific objectives, including any prespecified hypotheses	5 (15-16)
**Methods**			
Study design	#4	Present key elements of study design early in the paper	5(21-27)
Setting	#5	Describe the setting, locations, and relevant dates, including periods of recruitment, exposure, follow-up, and data collection	5 (30-36)
Eligibility criteria	#6a	Give the eligibility criteria, and the sources and methods of selection of participants. Describe methods of follow-up.	5 (30-36)
Eligibility criteria	#6b	For matched studies, give matching criteria and number of exposed and unexposed	NA
Variables	#7	Clearly define all outcomes, exposures, predictors, potential confounders, and effect modifiers. Give diagnostic criteria, if applicable	5 (39) – 6 (25)

For peer review only

Data sources / measurement	#8	For each variable of interest give sources of data and details of methods of assessment (measurement). Describe comparability of assessment methods if there is more than one group. Give information separately for for exposed and unexposed groups if applicable.	5 (39) – 6 (25)
Bias	#9	Describe any efforts to address potential sources of bias	5 (33-36)
Study size	#10	Explain how the study size was arrived at	5 (30-31)
Quantitative variables	#11	Explain how quantitative variables were handled in the analyses. If applicable, describe which groupings were chosen, and why	6 (28-36)
Statistical methods	#12a	Describe all statistical methods, including those used to control for confounding	6 (36) –7 (4)
Statistical methods	#12b	Describe any methods used to examine subgroups and interactions	NA
Statistical methods	#12c	Explain how missing data were addressed	7 (1-4)
Statistical methods	#12d	If applicable, explain how loss to follow-up was addressed	NA
Statistical methods	#12e	Describe any sensitivity analyses	7 (1-4)
**Results**			
Participants	#13a	Report numbers of individuals at each stage of study—eg numbers potentially eligible, examined for eligibility, confirmed eligible, included in the study, completing follow-up, and analysed. Give information separately for for exposed and unexposed groups if applicable.	Figure
Participants	#13b	Give reasons for non-participation at each stage	NA
Participants	#13c	Consider use of a flow diagram	Figure
Descriptive data	#14a	Give characteristics of study participants (eg demographic, clinical, social) and information on exposures and potential confounders. Give information separately for exposed and unexposed groups if applicable.	13
Descriptive data	#14b	Indicate number of participants with missing data for each variable of interest	13
Descriptive data	#14c	Summarise follow-up time (eg, average and total amount)	7 (20-23)
Outcome data	#15	Report numbers of outcome events or summary measures over time. Give information separately for exposed and unexposed groups if applicable.	15,16
Main results	#16a	Give unadjusted estimates and, if applicable, confounder-adjusted estimates and their precision (eg, 95% confidence interval). Make clear which	15,16

For peer review only

		confounders were adjusted for and why they were included	
Main results	#16b	Report category boundaries when continuous variables were categorized	15,16
Main results	#16c	If relevant, consider translating estimates of relative risk into absolute risk for a meaningful time period	NA
Other analyses	#17	Report other analyses done—e.g., analyses of subgroups and interactions, and sensitivity analyses	Supp tables 1,2
**Discussion**			
Key results	#18	Summarise key results with reference to study objectives	8 (5-16)
Limitations	#19	Discuss limitations of the study, taking into account sources of potential bias or imprecision. Discuss both direction and magnitude of any potential bias.	8 (19) – 10 (27)
Interpretation	#20	Give a cautious overall interpretation considering objectives, limitations, multiplicity of analyses, results from similar studies, and other relevant evidence.	8-10
Generalisability	#21	Discuss the generalisability (external validity) of the study results	9 (9-20)
**Other Information**			
Funding	#22	Give the source of funding and the role of the funders for the present study and, if applicable, for the original study on which the present article is based	1 (46)

The STROBE checklist is distributed under the terms of the Creative Commons Attribution License CC-BY. This checklist was completed on 20. October 2020 using https://www.goodreports.org/, a tool made by the EQUATOR Network in collaboration with Penelope.ai

For peer review only
